# RNA-*Seq *and molecular docking reveal multi-level pesticide resistance in the bed bug

**DOI:** 10.1186/1471-2164-13-6

**Published:** 2012-01-06

**Authors:** Praveen Mamidala, Asela J Wijeratne, Saranga Wijeratne, Karl Kornacker, Babu Sudhamalla, Loren J Rivera-Vega, Andrew Hoelmer, Tea Meulia, Susan C Jones, Omprakash Mittapalli

**Affiliations:** 1 Department of Entomology, The Ohio State University, Ohio Agricultural and Research Development Center, Wooster, OH 44691, USA; 2 Molecular and Cellular Imaging Center, The Ohio State University, Ohio Agricultural and Research Development Center, Wooster, OH 44691, USA; 3 Division of Sensory Biophysics, The Ohio State University, Columbus, OH 43210, USA; 4 School of Chemistry, University of Hyderabad, Hyderabad, AP 500046, India; 5 Department of Entomology, The Ohio State University, Columbus, OH 43210, USA

## Abstract

**Background:**

Bed bugs (*Cimex lectularius*) are hematophagous nocturnal parasites of humans that have attained high impact status due to their worldwide resurgence. The sudden and rampant resurgence of *C. lectularius *has been attributed to numerous factors including frequent international travel, narrower pest management practices, and insecticide resistance.

**Results:**

We performed a next-generation RNA sequencing (RNA-*Seq*) experiment to find differentially expressed genes between pesticide-resistant (PR) and pesticide-susceptible (PS) strains of *C. lectularius*. A reference transcriptome database of 51,492 expressed sequence tags (ESTs) was created by combining the databases derived from *de novo *assembled mRNA-*Seq *tags (30,404 ESTs) and our previous 454 pyrosequenced database (21,088 ESTs). The two-way GLMseq analysis revealed ~15,000 highly significant differentially expressed ESTs between the PR and PS strains. Among the top 5,000 differentially expressed ESTs, 109 putative defense genes (cuticular proteins, cytochrome P450s, antioxidant genes, ABC transporters, glutathione *S*-transferases, carboxylesterases and acetyl cholinesterase) involved in penetration resistance and metabolic resistance were identified. Tissue and development-specific expression of P450 CYP3 clan members showed high mRNA levels in the cuticle, Malpighian tubules, and midgut; and in early instar nymphs, respectively. Lastly, molecular modeling and docking of a candidate cytochrome P450 (CYP397A1V2) revealed the flexibility of the deduced protein to metabolize a broad range of insecticide substrates including DDT, deltamethrin, permethrin, and imidacloprid.

**Conclusions:**

We developed significant molecular resources for *C. lectularius *putatively involved in metabolic resistance as well as those participating in other modes of insecticide resistance. RNA-*Seq *profiles of PR strains combined with tissue-specific profiles and molecular docking revealed multi-level insecticide resistance in *C. lectularius*. Future research that is targeted towards RNA interference (RNAi) on the identified metabolic targets such as cytochrome P450s and cuticular proteins could lay the foundation for a better understanding of the genetic basis of insecticide resistance in *C. lectularius*.

## Background

*Cimex lectularius *(the bed bug), a hematophagous ectoparasite of humans, is now spreading at alarming rates across the globe [1-6]. The bites of these nocturnal blood feeders result in cutaneous manifestations, urticarial reactions, and occasionally anaphylaxis [7]. Further, scratching of bite sites promotes secondary bacterial infections [8]. Though the role of *C. lectularius *in disease transmission remains unclear, bed bugs are known to carry methicillin-resistant *Staphylococcus aureus *(MRSA) and vancomycin-resistant *Enterococcus faecium *(VRE) [9,10]. *C. lectularius *associated with MRSA and VRE strains possibly amplify infections in impoverished urban communities [10].

It has been more than half a century since *C. lectularius *first showed resistance to DDT [11,12]. The sudden resurgence of *C. lectularius *is purportedly due to increased resistance to broad-spectrum insecticides, changes in pest management practices, frequent international travel and passive dispersal (clothing, luggage and second-hand furniture) [13,14]. In the recent past, *C. lectularius *severely affected the hospitality industry, wherein some hotels were closed due to heavy infestations [15]. The lack of effective pest management tools for this blood-feeding insect has led to its successful establishment around the globe [8].

Insects develop resistance to insecticides through four modes: penetration resistance (thicker cuticle for decreased entry of insecticides), behavioral resistance (avoidance of the toxic compounds), target site resistance (knockdown resistance, *kdr*), and metabolic resistance (detoxification primarily through the action of cytochrome P450s, glutathione *S*-transferases [GSTs], and carboxylesterases) [16]. Among these four modes, target site resistance (*kdr*) has been well characterized in *C. lectularius *[17,18], but little to no knowledge exists regarding the other modes of resistance. Recent transcriptomic studies of *C. lectularius *have set the foundation for understanding the potential contribution of cytochrome P450s (metabolic resistance) in pesticide resistance [19,20]. However, a more comprehensive and/or global understanding of the genes involved in pesticide resistance and their "*modus operandi*" in *C. lectularius *are necessary for understanding the genetic factors establishing resistance in *C. lectularius *and for improving existing control strategies or devising new ones. The next-generation sequencing (NGS) methods via different platforms (Illumina Genome Analyzer, Applied Biosystems SOLiD, Helicos Biosciences Heliscope, Roche 454 Life Sciences) have revolutionized functional genomics research in non-model organisms [21-24]. Illumina deep sequencing (RNA sequencing, RNA-*Seq*) has emerged as a powerful tool for simultaneous transcriptome characterization and differential gene expression (DGE) analysis to better understand eco-physiological adaptations of insects [24-28].

In this study, we used RNA-*Seq *to compare and contrast pesticide-resistant (PR) and pesticide-susceptible (PS) strains of *C. lectularius*. As a result, we have significantly enriched the existing transcriptomic database of *C. lectularius *[19] and subsequently identified candidate genes putatively involved in insecticide resistance. Lastly, molecular docking was performed for a candidate CYP3 clan member (CYP397A1V2) to further determine the likely contribution of P450 mediated insecticide resistance in *C. lectularius*.

## Results and Discussion

### *De novo *assembly

**Figure 1:**
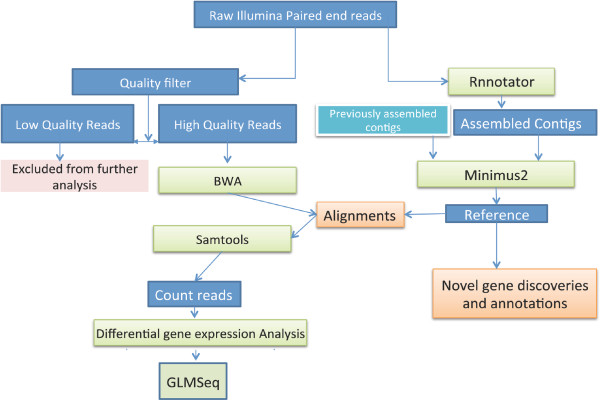
**Overview of work-flow for assembly of RNA-*Seq *reads and 454 contigs of *Cimex lectularius***.

**Figure 2:**
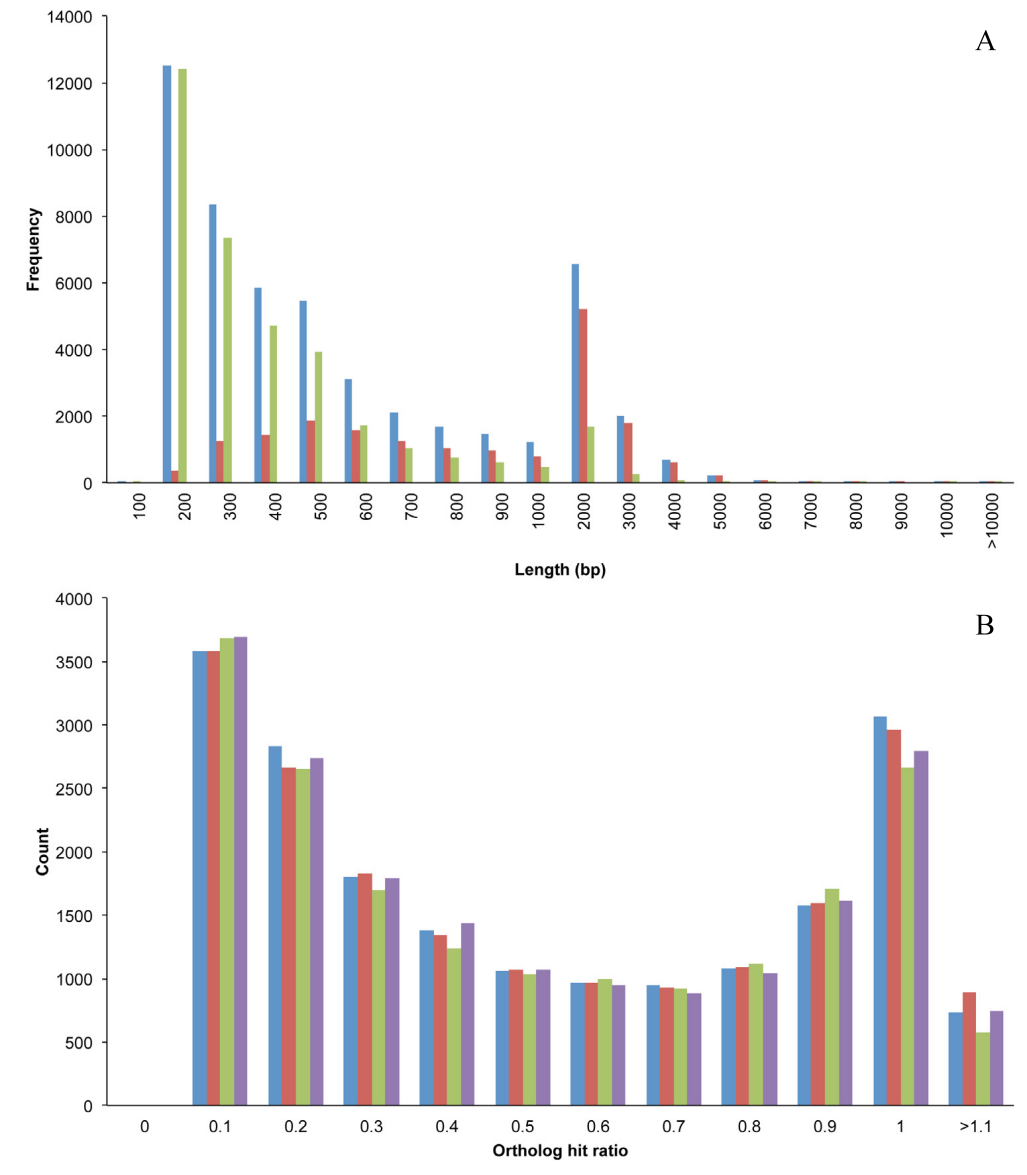
**Summary of *C. lectularius *transcriptome**. **(A) **Distribution of reference contigs (blue bars), supercontigs (red bars), and contigs (green bars) after assembly using CD Minimus. (B) Ortholog hit ratio (OHR) for assembled ESTs (*Aphis pisum- *blue bars, *Pediculus humanus corporis-*red bars; *Anopheles gambiae*-green bars; *Apis mellifera*-purple bars). An OHR of one indicates a fully assembled sequence whereas a value closer to zero suggests a poorly assembled sequence. Nearly half of the ESTs had an OHR more than 0.5.

We generated 62,107,336 and 64,214,910 reads for two PR strains and 72,748,924 and 72,400,340 reads for two PS strains (Table 1). An overview of the *de novo *assembly is shown in Figure 1. *De novo *assembly of the transcriptomes using an automated transcriptome assembly pipeline (see Methods) with 'PR' sample 1 and ''PS' sample 1 resulted in 34,385 (with N50 being 833 nt) and 46,412 (with N50 being 1064 nt) contigs, respectively (Table 1). Combining these contigs with previously assembled contigs (21,088; with N50 being 466 nt) [19] resulted in a total of 51,492 ESTs (after removal of redundant sequences) and these were used as reference sequences (Table 1, Figure 2A). The ESTcalc http://fgp.huck.psu.edu/NG_Sims/ngsim.pl estimated that the combined Illumina reads and 454 datasets would cover 90% of the bed bug transcriptome (Additional file 1).

**Table 1:** Summary statistics of Illumina and Roche 454 GS FLX reads of *Cimex lectularius*

	**PR1**^b^	PR2	PS1	PS2	**454**^c^	**FA**^d^
**Total number of reads**	6.40E+07	6.20E+07	7.30E+07	7.20E+07	N/A	N/A
**Number of reads after trimming for quality**^a^	6.30E+07	6.20E+07	7.20E+07	7.20E+07	N/A	N/A
**Number of reads used for assembly**	3.00E+07	N/A	4.00E+07	N/A	N/A	N/A
**Number of contigs**	34,385	N/A	46,412	N/A	21,088	51,492
**N50 value**	833	N/A	1064	N/A	456	1150
**Longest contig (bp)**	13476	N/A	20208	N/A	4699	21,222
**Number of reads mapped**	4.00E+07	5.00E+07	5.00E+07	6.00E+07	N/A	N/A
**Percentage of reads mapped to the FA**	63.00%	71%	85.00%	84.00%	N/A	N/A

^a^q < 20 and length < 20 nt were discarded; ^b^PR, pesticide-resistant; PS, pesticide-susceptible; ^c^Sequences were obtained from Bai et al. [19]; ^d^Final assembly (FA) was obtained by combining 454 sequences and assemblies from PR1 and PS1 samples.

The read variation number observed in the current study has been commonly associated with the Illumina platform as different lanes can produce different number of counts [25,27]. Other possible reasons for read variation include different percentages of poly A^+ ^in different samples, differential efficiency of conversion of RNA to cDNA, size or GC composition variation among libraries (smaller and/or lower GC content amplicons amplify more efficiently during PCR for library quantitation). All these factors may contribute to slight variations in the estimation of the library concentration that is applied onto the flow-cell, finally resulting in differences in cluster densities. The coverage and number of reads (i.e., depth of sequencing) obtained in this study far exceeds that of another recently published article on the *C. lectularius *transcriptome involving differential expression between susceptible and resistant populations [20].

To determine the completeness of our final assembly, each of the derived assembled sequences was compared with its putative ortholog from *Acyrthosiphon pisum, Anopheles gambiae, Drosophila melanogaster*, and *Pediculus humanus corporis*. Nearly, 33-35% of the assembled sequences exceeded a 0.8 ortholog hit ratio and 48-50% had more than a 0.5 ortholog hit ratio (Figure 2B). An ortholog ratio of one indicates a fully assembled transcriptome, while a value close to zero indicates a poor assembly [29]. The current transcriptome characterization resulted in a 23-fold enrichment of the existing EST database of *C. lectularius *(1,971 ESTs http://www.ncbi.nlm.nih.gov accessed on 25^th ^August 2011) which allowed fine characterization of the *C. lectularius *transcriptome as has been observed in many non-model insect species [24-28].

### Comparative genomics

**Figure 3:**
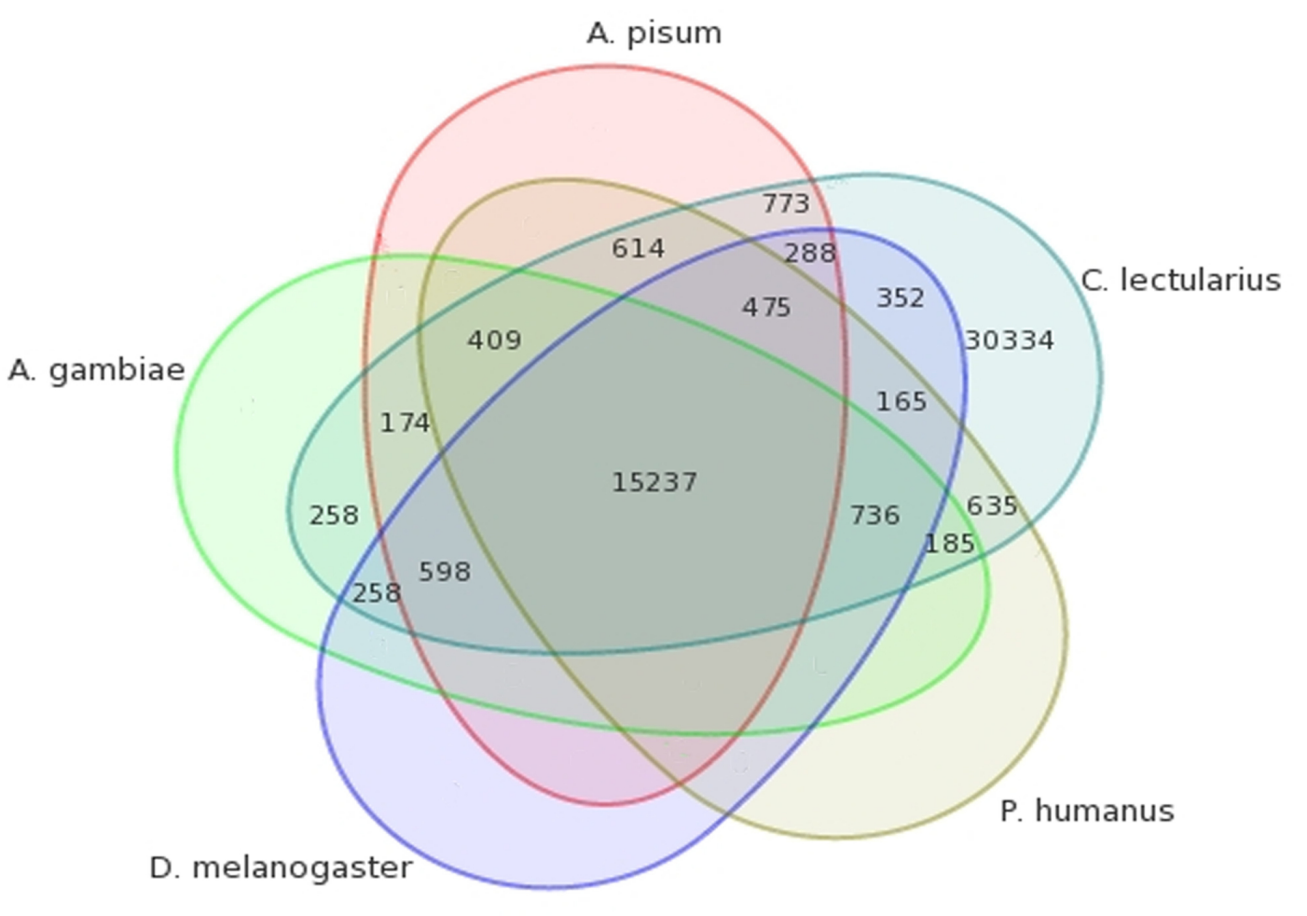
**A five-way Venn diagram showing the comparisons of assembled *Cimex lectularius *sequences with protein sequences of *Acrythosiphon pisum, Anopheles gambiae, Drosophila melanogaster *and *Pediculus humanus corporis***.

About 43% (21,908/51,492) of the *C. lectularius *ESTs had one or more hits to protein sequences in the non-redundant (nr) protein database http://www.ncbi.nlm.nih.gov with the remaining sequences (57%) being transcripts of unknown function (TUF). This is in agreement with our previous 454 EST datasets of *C. lectularius*, wherein we reported a similar percentage (45.2%) of EST hits with the nr protein database [19]. The majority of TUF sequences might be due to novel transcripts or genes whose biological functions are not assigned as has been observed in other transcriptomic studies [19,25-27]. The top BLAST hits of the *C. lectularius *ESTs showed majority hits to insects (65.45%) and non-insect eukaryotes (32.82%); there were a few hits (1.73%) to members of bacteria (Additional file 2). This trend in top BLAST hits is consistent with other NGS datasets reported for insects [19,30,31]. Similarity searches between *C. lectularius *ESTs and protein sequences of *Ac. pisum, A. gambiae, D. melanogaster *and *P. humanus corporis *revealed an overall 41% similarity (21,157/51,492), with the highest number of hits to *Ac. pisum *(36%; 18,568/51,492) and *P. humanus corporis *(35.8%; 18,456/51,492) (Figure 3). This similarity of *C. lectularius *with *Ac. pisum *and *P. humanus corporis *might be due to their phylogenetic relatedness.

### Functional annotation

A total of 15,540 out of the 51,492 ESTs (reference sequences) were annotated and Gene Ontology (GO) terms were found to be distributed in a wide variety of functional categories (Additional file 3). Annotation with the *D. melanogaster *genome revealed a similar distribution of GO categories with *C. lectularius *showing no notable bias towards any category. The majority of the ESTs annotated with biological processes were involved in development and biological regulation, while the ESTs annotated with molecular function revealed high catalytic and binding activity (Additional file 3). A number of putative viral sequences were represented in the GO terms for biological processes (viral life cycle) and cellular component (viroids) (Additional file 3 and Additional file 4). Though viral transmission by *C. lectularius *is still not clear, the Hepatitis B virus (HBV) has frequently been detected in wild *C. lectularius *[7]. Future studies are required to both validate these viral sequences and determine their biological relevance within the *C. lectularius *genome. The assigned metabolic pathways revealed a high number of sequences (1,557/8,159) to be involved in synthesis of carbohydrates, proteins, lipids and nucleotides (Additional file 5). These predicted pathways together with gene annotation will better help in revealing gene function in *C. lectularius *[19].

### Differential gene expression

RNA-*Seq *libraries in the current study were constructed from paired biological replicates of resistant and susceptible RNA samples of *C. lectularius*. The four RNA samples were processed in two batches: Batch-1 (Resistant-1, Susceptible-1) and Batch-2 (Resistant-2, Susceptible -2). Hence, we applied GLMseq (a custom R-script for fitting a two-way Generalized Linear Model) analysis to the four un-normalized RNA-*Seq *counts at each EST. To find differentially expressed ESTs, Illumina counts of all four populations (PS1, PS2, PR1and PR2, refer to the methods section for more details) were aligned to the assembled reference database which resulted in ~15,000 highly significant differentially expressed ESTs (P < 0.005). All differentially expressed ESTs, along with their Absolute Log2 Fold Change Adjusted (ALFCA) and their description based on BLAST hits, are presented in Additional file 6.

The primary objective of GLMseq analysis was to determine the amount and significance of the main resistance effects, setting aside any secondary batch effects due to transcript-specific interactions between batch effects and resistance effects. Currently available methods for RNA-*Seq *data analysis, such as DESeq, are limited to one-way analysis of replicate groups and consequently cannot correctly handle two-way interactions. Therefore, a custom R-script (GLMseq) was used to fit a two-way generalized linear model consisting of a main resistance effect and a secondary interaction effect.

### GO analysis of differentially expressed ESTs

**Figure 4:**
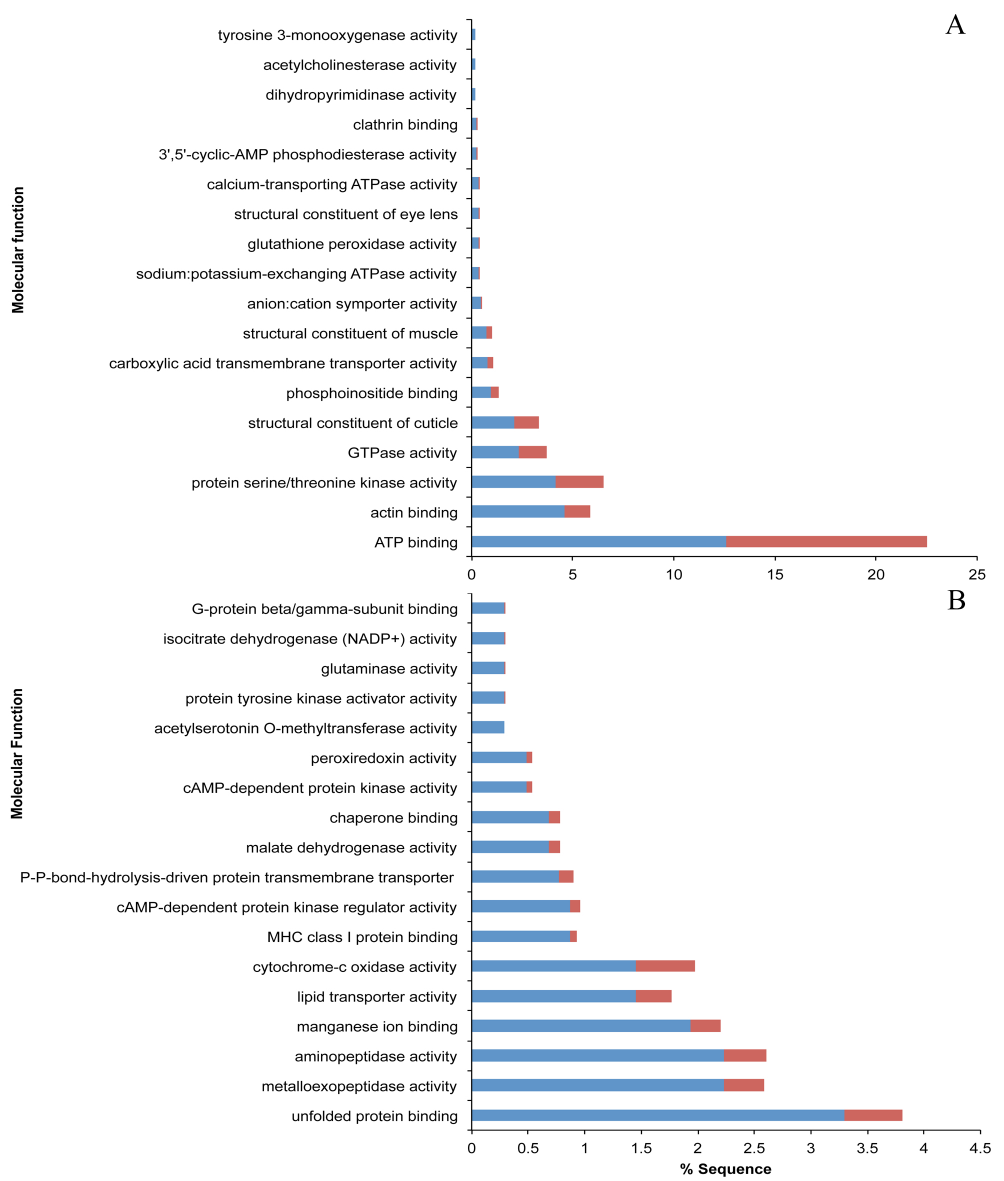
**Gene Ontology (GO) term enrichment analyses**. (A) Overrepresented GO terms for ESTs that are up-regulated in pesticide-resistant strain of *Cimex lectularius*. (B) Overrepresented GO terms for ESTs that are down-regulated in pesticide-resistant strain of *C. lectularius*. (Test: up- or down-regulated top 5,000 ESTs in blue bars; Reference: All annotated ESTs without test group represented in red bars).

GO enrichment analysis was performed for the top 5,000 differentially expressed ESTs obtained in GLMseq and all of the molecular function, biological process and cellular components are shown in Additional file 7. We focused on molecular function in order to identify potential categories of genes that are associated with pesticide resistance, such as have been revealed in other insect studies [32]. Of the top 5,000 genes about 52.62% (2,631) were up-regulated and 47.38% (2,369) were down-regulated in the PR strains. Among these, 19.86% (993/5,000) of the ESTs had no known function (transcripts of unknown function; TUFs) (Additional file 7). For the up-regulated cluster of differentially expressed ESTs, some of the enriched GO terms for molecular function included ATP binding, actin binding, serine/threonine kinase activity, structural constituent of cuticle, etc. (Figure 4A). The enriched GO terms for actin binding in PR strains of *C. lectularius *might suggest their potential involvement in cytoskeletal networks and cellular mechanical integrity as observed in other pyrethroid-resistant insect strains [33].

### Detoxification enrichment

We found enriched ATP binding and glutathione peroxidase-associated GO terms in the up-regulated cluster of differentially expressed ESTs suggesting the involvement of other detoxification genes such as ATP binding cassette (ABC) transporters and quenchers of reactive oxygen species (ROS) (Figure 4A), which is in agreement with other insect studies [34,35]. High occurrences of P450s (29) in the top 5,000 differentially expressed ESTs of the *C. lectularius *PR strain suggest their putative role in metabolic resistance (Table 2). Insect P450s are one of the key players in detoxification and metabolism of a broad range of toxins including plant-synthesized and synthetic compounds [36]. The top differentially expressed ESTs were found in all the major clades of P450s (25 CYP3, 2 CYP4, and 2 mitochondrial CYP clan members) (Additional file 8). Among these, the up-regulated differentials in PR strains (65.51%) were exclusively CYP3 clan members, which are thought to be the primary P450s involved in detoxification of xeno- and endobiotics in insects [37]. Besides the CYP3 clan of P450s, CYP4 and mitochondrial CYP clan members are also thought to participate in insecticide resistance [38]. However, in the top 5,000 differentials, the CYP4 and mitochondrial P450 members were found to be down-regulated (Additional file 8).

**Table 2:** Summary of differentially expressed genes involved in insecticide resistance of Cimex lectularius

Mode of resistance	Number	Number in the top 5,000 differentials		qRT-PCR validated
			
		Up-regulated	Down-regulated	
***Metabolic resistance***				
Cytochrome P450	102	19	10	BB_Contig_103, 18015, 19601, 22399
ABC transporters	27	8	2	BB_Contig_1346
GSTs	18	3	1	NA
Catalase	13	2	0	NA
Superoxide dismutase	6	1	1	BB_Contig_49102
Carboxylesterases	6	2	0	NA
Glutathione peroxidase	7	3	0	NA
***Penetration resistance***				
Cuticular proteins	247	46	8	BB_Contig_1762,1766, 17694, 21630, 48951
***Insecticidal targets***				
Acetylcholinesterases	6	3	0	BB_Contig_3653

### Cuticular enrichment

The enriched GO terms for actin binding, structural constitution of cuticle, and structural constituent of muscle in PR strains of *C. lectularius *could imply the strengthening of structural components such as the cuticle and midgut-associated structures. Indeed among the up-regulated cluster of ESTs, we found a high number (46) of transcripts encoding for cuticular proteins (Table 2). Cuticular proteins are major components of insect cuticle. The constitutive or induced expression of such proteins potentially establishes penetration resistance in *C. lectularius*, as observed in other insect systems [39]. It is interesting to observe that only 8 transcripts encoding cuticular proteins were found in the down-regulated differential cluster in PR strains of *C. lectularius *(Table 2).

For the down-regulated cluster of differentially expressed ESTs, the most enriched GO terms included metalloexopeptidase, aminopeptidase activities, manganese ion binding, lipid transporter activity, etc. (Figure 4B). These trends could imply the transcriptomic adjustments within *C. lectularius *upon encountering insecticides. While Adelman et al. [20] attribute multi-level insecticide resistance of bed bugs primarily toward the activity of metabolic genes (cytochrome P450, carboxylesterases and GSTs) plus *kdr *mutations, their analysis failed to identify major players involved in penetration resistance (e.g., cuticular proteins). Further, in addition to the metabolic genes identified in their study, our unbiased/global approach identified other key players of metabolic resistance such as ABC transporters and antioxidant genes (Table 2).

We also screened the ESTs that were uniquely expressed in susceptible and resistant populations of *C. lectularius*, wherein a high number of ESTs (77) were found in PS strains and a few (7 ESTs) in PR strains (Additional File 9). In both cases, a majority of the ESTs were TUFs (85.71% in PR and 74.02% in PS strains). At the current time, it is too speculative to explain the uniquely expressed ESTs encoding for cathepsin in PR strains and RP45 and keratin-associated proteins among PS strains (Additional file 9).

### RNA-*Seq *validation of candidate genes

To validate the expression profiles obtained through GLMseq analysis, quantitative real time RT-PCR (qRT-PCR) was performed on 12 selected candidate genes (cuticular proteins, CYP3 and mitochondrial P450 clan members, ABC transporter, superoxide dismutase, and acetylcholinesterase) belonging to the top differential cluster (Additional file 10). Except for one of the candidate genes profiled (mitochondrial CYP member, CYP 301A2), the qRT-PCR results correlated with the GLMseq profiles (Additional file 10). The expression patterns of CYP301A2 of *C. lectularius *are in corroboration with DDT-resistant and DDT-susceptible fruit flies (*D. melanogaster*), wherein no appreciable differences of CYP301A1 transcript levels were reported between the susceptible and resistant flies. Further, DDT treatment did not induce the expression levels of CYP301A1 [40]. The mitochondrial P450 clan members are exclusively found in animals with conserved and diversified groups potentially involved in physiological and detoxification processes in insects [41,42]. Though these clan members have been well documented to detoxify insecticides and plant allelochemicals [42-44], the CYP301A2 of *C. lectularius *might plausibly also participate in the synthesis of 20-hydroxyecdysone (20-HE) as observed in other insect studies [45,46].

The PR strains of *C. lectularius *displayed higher transcript levels for all of the assayed cuticular proteins (larval cuticle protein [LCP], pupal cuticle protein [PCP], chitin synthase [CHS], chitin deacetylase [CDA] and cuticular protein analogous to peritrophin [CPAP]) and are consistent with the RNA-*Seq *profiles (Additional file 10). The higher expression of LCP and PCP in PR strains could contribute to increased pyrethroid resistance [47]. In addition to their role in other physiological processes, insects commonly up-regulate transcript levels of cuticular proteins to reduce the penetration rate of insecticides [39,47,48]. Higher transcript levels for CDA and CHS could contribute altered chitin and chitosan ratios in cuticle, which influences insect survival [49]. Co-expression of the CHS and CDA transcripts might potentially reduce the entry of xenobiotic compounds into the insect body. The higher expression of CPAP in PR strains (Additional file 10) indicates a possible role of the peritrophic membrane (gut) in sequestering, detoxifying ingested xenobiotics in addition to its role in peritrophic membrane formation and protection from invasive parasites [50,51].

All three cytochrome P450s of CYP3 clan members (CYP397A1V2, putative CYP6A2 and CYP6A13) revealed higher mRNA levels in the PR strains (Additional file 10). This is in agreement with other insect systems showing resistance to DDT or pyrethroids [52]. The transcript levels of a superoxide dismutase and an ABC transporter were also found to be higher in the PR strains (Additional file 10), which suggests that these proteins are potentially involved in the elimination and efflux of intracellular toxins thereby reducing their interaction with intracellular targets [35,53]. The transcripts encoding acetylcholinesterase (insecticide target protein) showed significantly higher expression in the PR strains (Additional file 10) possibly resulting in increased production of acetylcholinesterase to minimize the toxic effects of insecticides [54]. The current qRT-PCR profiles of three P450s and an ABC transporter together with our previous findings of a Phase II protein (GST) [19] suggest a phase-wise detoxification of xenobiotic compounds in *C. lectularius*. Further, the qRT-PCR profiles also validate our GLMseq analysis employed for generating the differentially expressed ESTs.

### Tissue and development-specific expression of CYP397A1V2 and CYP6A13

Given our interest in the role of P450s in metabolic resistance, we further profiled the transcript levels of CYP397A1V2 and CYP6A13 in different tissues (cuticle, midgut and Malpighian tubules) of adults and different developmental stages (early and late instar nymphs and adults) of the PR and PS strains. Both genes showed lesser transcript levels in PS strains, therefore the PS strains were used as a calibrator to determine fold change in tissues and developmental stages of the PR samples.

**Figure 5:**
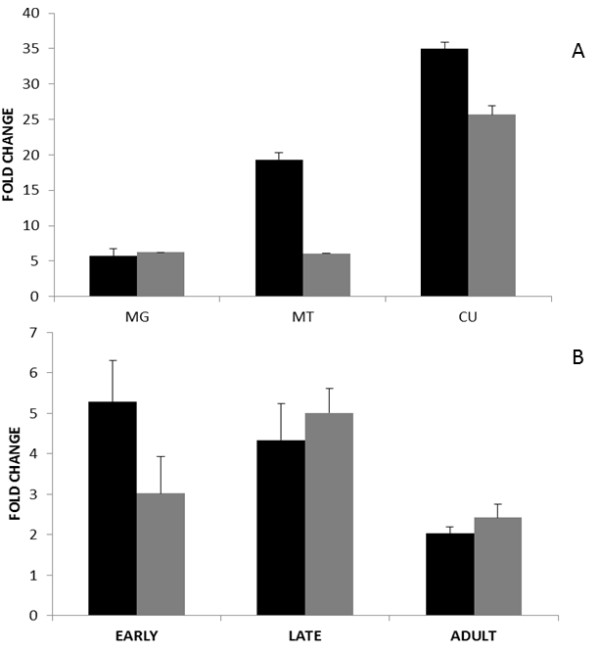
**qRT-PCR analysis of CYP397A1V2 and CYP6A13 in *Cimex lectularius***. (A) mRNA levels of CYP397A1V2 (black bars) and CYP6A13 (grey bars) in tissues of *C. lectularius*. Tissues assayed included cuticle (CU), Malpighian tubules (MT), and midgut (MG). Tissues samples of pesticide susceptible strains were taken as calibrator to calculate fold change. (B) qRT-PCR analysis of CYP397A1V2 (black bars) and CYP6A13 (grey bars) in *C. lectularius *early instar nymphs, late instar nymphs and adults. The pesticide susceptible strains were taken as calibrator to calculate fold change. A *C. lectularius*-specific RPL-18 was used as an internal control. Standard error of the mean of three biological replicates and two technical replicates (within each biological replicate) is represented by the error bars.

An interesting finding of this study was the peak mRNA levels of CYP397A1V2 in the cuticle (Figure 5A) and relatively high mRNA levels in the Malpighian tubules and midgut. This suggests the possible function of P450-mediated detoxification of pesticides in the cuticle of *C. lectularius *and/or its participation in other physiological functions. However, these findings need further functional validation. High transcript levels of both the P450s (CYP397A1V2 and CYP6A13) in the Malpighian tubules could also correlate with detoxification given their role in metabolism and excretion of endogenous solutes and xenobiotics [55]. The role of P450s in the insect midgut has been well established in detoxification as well as possible pheromone synthesis [56].

The qRT-PCR analysis of CYP397A1V2 and CYP6A13 showed higher mRNA levels in all developmental stages of PR compared to PS strains, specifically peak levels in early instar nymphs for CYP397A1V2 and late instar nymphs for CYP6A13 (Figure 5B). Our findings correlate with those in other insect systems and therefore suggest the up-regulation of P450s in *C. lectularius *to efficiently detoxify a broad range of toxic substrates at different tissue interfaces and during development [19,42,56-58].

The expression of CYP397A1V2 transcripts in adults are in agreement with recent transcriptomic studies of *C. lectularius *[20]. However, our results clearly demonstrate the broader appearance of key metabolic players of both penetration resistance as well as metabolic resistance compared to Adelman et al. [20].

### Homology modeling

**Figure 6:**
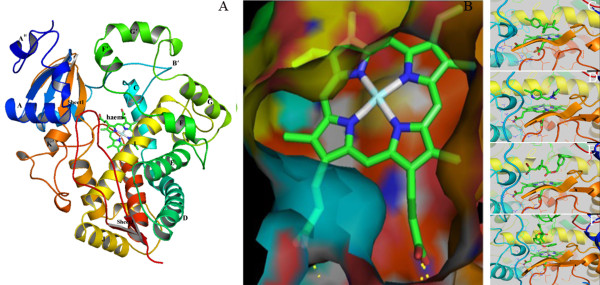
**Molecular modeling and docking of CYP397A1V2 with substrate insecticides**. (A) Cartoon representation of the homology model of CYP397A1V2. The cartoon model of CYP397A1V2 is colored using a blue to red gradient from N-terminus to C-terminus. The model was created with MODELLER 9v8 using human cytochrome P450 CYP3A4 as a template. (B) Clipped view of the 'V' shaped molecular surface of the active site cavity. (C-F) Insecticide substrates docked into the binding site of CYP397A1V2 represented as a cartoon model: DDT (C), imidacloprid (D), deltamethrin (E), and permethrin (F), depicted in a stick model (light green). CYP397A1V2 residues interacting with ligand rendered as the line model. The images were made using Pymol pymol.sourceforge.net.

Given the unique tissue-specific expression profiles of CYP397A1V2 in PR strains, the full-length sequence of CYP397A1V2 was obtained, which revealed all the characteristic features of a CYP3 clan member (Additional file 11). A three-dimensional model for CYP397A1V2 was generated using human cytochrome P450 CYP3A4 (PDB code: 1WOE) as the template (Figure 6A and Additional file 12). During the homology modeling process, an iron-heme molecule was included and connected by creating a covalent bond between the heme's iron atom and the sulfur atom of conserved cysteine (Cys^432^) with the propionates of the heme interacting with the side chains of Trp^127^, Arg^131^, and Arg^369^. The CYP397A1V2 model displayed Phenylalanine-cluster (Phe^120^, Phe^121^, Phe^138 ^and Phe^297^) above the active site region, with the aromatic side chains stacking against each other to form a prominent hydrophobic core. The Ramachandran plot for CYP397A1V2 showed that approximately 94% of all residues were within the generously allowed region and 1.7% of residues were in the disallowed region (Additional file 13). Thus, the CYP397A1V2 model confirms to the conserved structural folding of P450s, with some unique features including: i) the hydrophobic region was located around the loop (following helix A" and G'-G" helices), ii) the region following helix F appeared shorter compared to other P450s and includes a ordered stretch of polypeptide chain that does not confirm to any secondary structural motif (Figure 6A), and iii) the catalytic pocket of the CYP397A1V2 displayed a smaller volume compared to other insect P450 proteins (Figure 6B).

### Molecular docking

**Figure 7:**
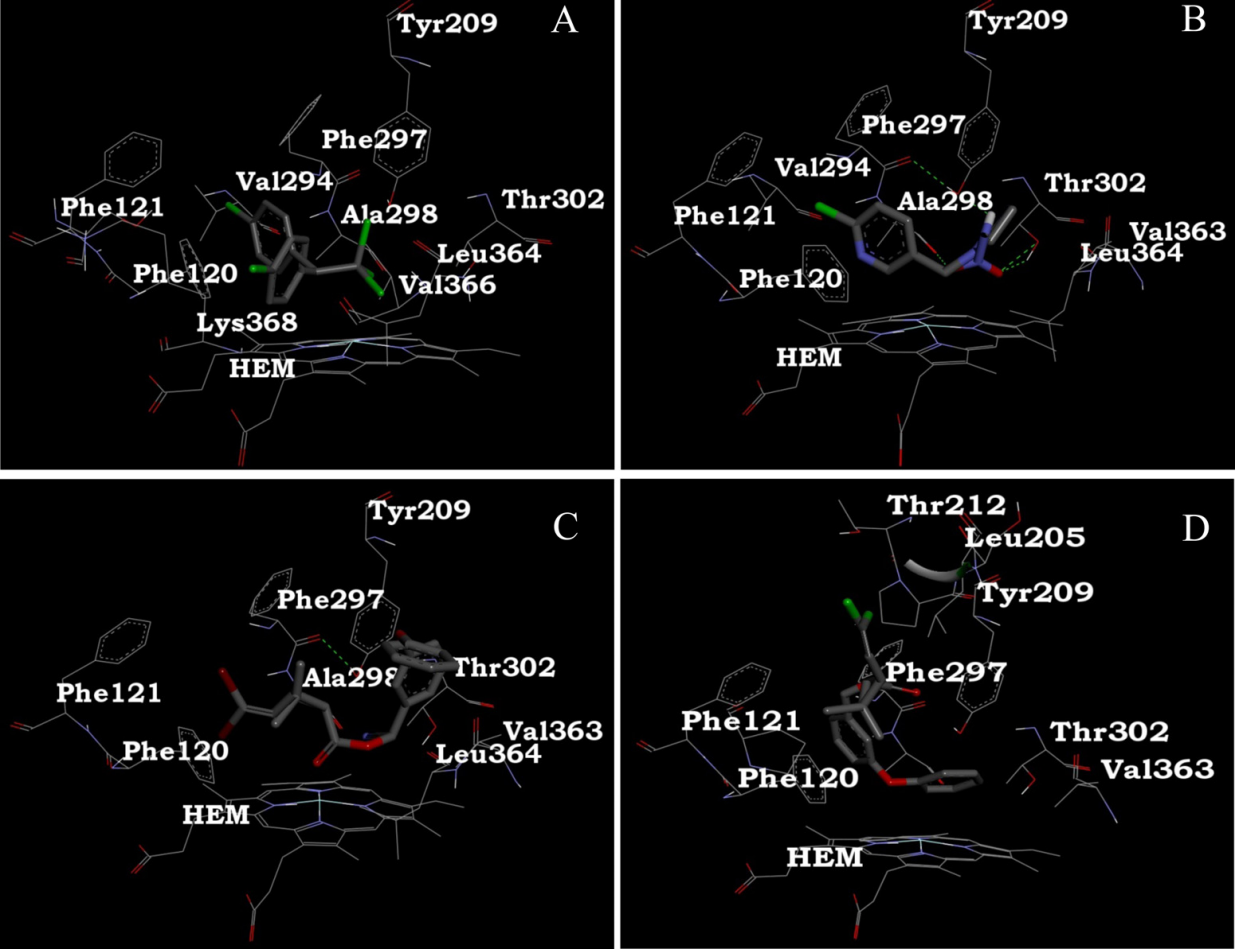
**Catalytic pockets in the CYP397A1V2 with heme and amino acid residues**. (A) CYP397A1V2-DDT complex. (B) CYP397A1V2-imidacloprid complex. (C) CYP397A1V2-deltamethrin complex (D) CYP397A1V2-permethrin complex. The biochemical properties of amino acids are represented in various colors.

We used the program Autodock to examine the binding mode of CYP397A1V2 with various insecticides (DDT, imidacloprid, deltamethrin, permethrin and diazinon), any of which *C. lectularius *may encounter worldwide. The binding constants and free energy change (Additional file 14) revealed DDT, permethrin, deltamethrin and imidacloprid as potential substrates due to their tight-fit into the active site ('V'- shaped hydrophobic cavity) of CYP397A1V2 (Figure 6C-F). The moiety of DDT and permethrin molecules was located within the binding pocket, and they were adjacent to hydrophobic residues of the Phenylalanine cluster, Val^294^, Ala^298^, Lys^368^, Val^366 ^and Leu^364 ^of the derived model CYP397A1V2 (Figure 7A and 7D). Thus, the interaction of DDT and permethrin with CYP397A1V2 appears to be dominated by mainly hydrophobic interactions. The insecticide substrates deltamethrin and imidacloprid were also fully enclosed by receptor residues in the cavity of CYP397A1V2 and were positioned well within the network of hydrophobic as well as hydrophilic residues. In the case of deltamethrin and imidacloprid, the common receptor residues Tyr^209^, Ala^298 ^and Thr^302 ^contributed to the formation of hydrogen bonds between the CYP397A1V2-deltamethrin and CYP397A1V2-imidacloprid complex (Figure 7B and 7C).

DDT and pyrethroids (permethrin and deltamethrin) are preferential sodium channel modulators in insects [59]. The frequent exposures to DDT, an early generation insecticide during the last century may have altered the development of resistance in *C. lectularius *to the next generation insecticides through a phenomenon called cross resistance or multi-level resistance [60]. The latter scenario might be the likely phenomenon associated with modern *C. lectularius*. Our docking effort on CYP397A1V2 supports this hypothesis. Similar observations were found in *Papilio glaucus*, wherein *Pc*CYP6B4 had the ability to metabolize a broad range of substrates [61]. The unlikely binding of diazinon with CYP397A1V2 suggests potential involvement of other P450s or metabolic proteins that interact with polar compounds.

### Validation for *kdr *mutation

We also examined the PR and PS strains for mutations in the voltage-gated sodium ion channels, as this is a well-characterized *kdr *mechanism found in *C. lectularius *[17]. All three PR strains showed both mutations (V419L and L925I) (Additional file 15). Although the PS1 and PS2 strains did not carry any of these mutations, the PS3 strain showed a single mutation at V419L (Additional file 15). These results further suggest that resistance in the PR strains could be attributed to multiple mechanisms of resistance including mutations as well as penetration and metabolic resistance.

## Conclusions

In summary, we have developed significant molecular resources for *C. lectularius *and have identified several candidate genes potentially involved in different phases of insecticide metabolism as well as those participating in other modes of insecticide resistance in this species. Our GLMseq approach further revealed significant differentially expressed ESTs across biologically variant samples. Specifically, the high occurrence of up-regulated cuticular proteins and the expression patterns of P450s in cuticular tissue might represent unique sites for penetration resistance as well as metabolic resistance in *C. lectularius*. Molecular modeling and docking studies revealed the potential of P450s to metabolize multiple insecticides in *C. lectularius*. Future functional studies (RNA interference) on cuticular and P450 proteins could lay the foundation for identifying hot-spots for insecticide resistance in *C. lectularius*, which could provide the basis for developing effective management strategies.

## Methods

### Insect material for RNA-*Seq*

Six populations (3 pesticide-susceptible strains--Harlan 1, Harlan 2, and FV strain hereinafter referred to as PS1, PS2 and PS3; 3 pesticide-resistant strains collected during 2010 from three residences in different zip codes in Columbus, OH, hereinafter referred to as PR1, PR2 and PR3) of *C. lectularius *were used in the current study. Bed bug populations were maintained under ambient conditions in the laboratory (~22 ± 1.22°C and 35 ± 6% RH). For each bed bug population, all stages and representatives of multiple generations were housed together on filter paper strips contained in a glass jar (13 cm high by 7 cm diameter) (narrow-mouth Mason pint jar, Ball Corp., Broomfield, CO) with an organza fabric and filter paper covering held in place using a screw-on ring. Multiple jars were needed for large populations. Each bed bug population was fed *in situ *approximately every 2 weeks on heparinized chicken blood using the Hemotek 5W1 membrane feeding system for blood-sucking insects (Discovery Workshops, Accrington, United Kingdom) with Parafilm^® ^as the membrane.

The resistance status of bed bug populations was assessed using a discriminating dose adapted from Romero et al. [13]. The Harlan strain was included as a standard susceptible strain given its long-term laboratory rearing status (since 1973). For this test, 10 adult bed bugs from each test population were placed on filter paper discs treated at a rate of 0.13 mg/cm^2 ^with technical grade deltamethrin (99% purity, Chem Service, Westchester, PA) dissolved in acetone. Acetone-treated filter paper was used as a control. Each population was replicated in triplicate. The condition of the bugs assessed after 24 h exposure to the treated filter paper. At 24 h, 100% of susceptible bed bugs (Harlan strains [PS1, PS2]) were either moribund or dead after exposure to technical deltamethrin, whereas resistant bed bugs (PR1, PR2, PR3) did not show any signs of intoxication. Preliminary resistance testing with PS3 indicated that despite having a known *kdr *mutation (V419L) (Additional file 15), it was succeptible to dried residues of both lambda-cyhalothrin and deltamethrin at or near the label rate.

### RNA isolation and Illumina Paired End (PE) library preparation

Total RNA was extracted separately from each individual (8 adults per population of PS1, PS2, PR1 and PR2) using TRIzol (Invitrogen) and pooled before conducting a quality check (Thermo Scientific Nanodrop 2000 and Agilent Bioanalyzer, Ohio State University, Columbus, OH, USA). Illumina high-throughput sequencing was done using the GAII platform at the Molecular and Cellular Imaging Center, Ohio Agriculture Research and Development Center, Wooster, OH, USA. Samples were prepared for Illumina GA sequencing using the PE library preparation kit (Catalogue Number PE-102-1001, Illumina, San Diego, CA, USA) and TruSeq RNA Sample Prep Kits (Catalogue Number FC-122-1001, Illumina, San Diego, CA, USA) per manufacturer instructions. Briefly, Sera-mag Magnetic Oligo (dT) beads were used for the poly (A) RNA enrichment and divalent cations were used to fragment the purified mRNA (100-400 bp) by heating the reaction mixture at 94°C for 5 minutes. The fragmented RNA was used for double strand synthesis followed by cDNA synthesis. The DNA was end-repaired and phosphorylated using T4 DNA polymerase, Klenow DNA polymerase and T4 PNK. These fragments were 3' adenylated using Klenow Exo- (3' to 5' exo minus) and Illumina PE adapters were ligated using DNA Ligase. To select appropriate size (for PE library preparation kit) and to eliminate unligated adapters, the adapter ligated products were purified on a 2% TAE-agarose gel (Certified Low-Range Ultra Agarose, Cat. No., 161-3107, Life Science Research, Hercules, CA, USA). cDNA fragments with 200 ± 25 bp were cut from the gel and purified using Qiagen gel purification kit (Cat. No. 28704, Qiagen, Valencia, CA, USA). For the Truseq RNA sample prep kit, size selection and purification from unligated adapter were done using Agencourt AMPure XP beads (Catalogue Number: A63881 Beckman Coulter Genomics Danvers, MA, USA). To enrich adapter ligated fragments and to add additional sequences necessary for flowcell binding, 15 rounds of PCR were performed using Illumina PE 1.0 and PE 2.0 primers (for the PE library preparation kit). The libraries were validated on an Agilent Technologies 2100 Bioanalyzer using the Agilent DNA 1000 chip kit and quantified using quantitative PCR with PhiX sequencing control as a standard (Cat. No. CT-901-2001 Illumina, San Diego, CA, USA). These libraries were sequenced using an Illumina GA II sequencer.

### Raw data analysis and *de novo *assembly

To eliminate low quality nucleotides, raw Illumina reads were trimmed using a custom Perl script with windowed adaptive trimming (Phred quality threshold of 20 and minimum read length of 20 nt). To do the *de novo *assembly, the trimmed reads from PR sample 1 and PS sample 1 were separately fed into Rnnotator, automated *de novo *RNAseq assembly pipeline [62], to remove duplicates and erroneous sequences. Multiple rounds on velvet assemblies then were performed using different hash values to account for the different sequencing depth for different transcripts [62]. Resulting contigs were merged using Minumus2 from the AMOS package [62,63]. These were combined with previously published *C. lectularius *454 sequences [19] for better coverage and quality using Minumus2 from the AMOS package to make the final reference contigs.

### Validation and annotation of assembled contigs

To validate the unigenes, we used three criteria: BLASTx searches (*E *value < 10^-3^) between unigenes and the NCBI non-redundant protein (nr) database http://www.ncbi.nlm.nih.gov; BLASTx searches (*E *value < 10^-3^) between unigenes and several insect species with genome sequence information (*A. pisum, A. gambiae, D. melanogaster, P. humanus*); and prediction of putative open reading frames (ORFs) using http://www.scbi.uma.es/cgi-bin/full-lengther/full-lengther_login.cgi. The ortholog hit ratio was calculated using custom python script [64]. To annotate the unigenes, BLASTx searches (*E *value < 10^-3^) were performed between nr and unigenes. BLAST results were imported to Blast2GO program for further annotation of the unigenes [65]. After the mapping step, those gene ontology (GO) terms with E value < 1E-3, annotation cut-off > 45, and GO weight > 5 were used for annotation. To find the pathways in which putative peptides of the unigenes are involved, analysis of Kyoto Encyclopedia of Genes and Genomes (KEGG) was performed using Blast2GO [66]. To find enriched GO terms, enrichment analysis (Fisher's Exact Test) tool in Blast2GO software was used with term filter value below 0.05, term filter mode "FDR" and two-tailed test. To categorize the GO terms into different GO categories, a web-based tool, CateGOrizer with "Aqua" tool was used and these categories were compared to a precomputed GO terms from *Drosophila melanogaster *[67,68].

### Differential gene expression analyses

Differential gene expression analysis among PR and PS samples was performed using two-way GLMseq, a custom R-script for fitting a two-way Generalized Linear Model to the four un-normalized RNA-*Seq *counts at each contig. The GLM function in R was used to estimate and test both the replicate effect and the resistance effect for each quartet of Poisson counts, with offsets determined by the total mapped reads for each sample. This model-free method makes no assumptions about the four unknown Poisson parameters and requires no pseudo-reference for the calculation of p-values.

The model treated each count as an independently measured Poisson variate with offset determined by library size. The examples below illustrate the effectiveness of GLMseq for setting aside secondary batch effects: In example A (Contig_18664), the raw counts for Batch-1 (4, 4) and Batch-2 (75359, 22) clearly suggest a large interaction effect (Additional file 16). In this case the GLMseq estimate of the main Resistant/Susceptible fold-change was 1.14, with a two-sided p-value of 0.85, indicating the absence of a significant resistance effect. In example B (Contig_27586), the raw counts for Batch-1 (18, 4) and Batch-2 (15041, 527) clearly suggest a positive resistance effect (Additional file 16). In this case the GLMseq estimate of the main Resistant/Susceptible fold-change was 5.15, with a two-sided p-value of 0.003, indicating the presence of a significant positive resistance effect. In both examples, naïve analysis of the pooled Resistant and Susceptible counts would have mistakenly assigned much lower p-values to the estimated fold-changes.

For finding uniquely expressed genes in PR and PS, the read counts were normalized using EDASeq method, which performs normalization for sequence length and GC content for intra and inter samples [69]. We considered ESTs with less than 10 short reads aligned in both biological replicates as not reliably expressed; for the expressed genes, we used more than 100 short reads aligned in both biological replicates as an arbitrary cut off.

### cDNA preparation for quantitative PCR analysis

Individuals from the six strains (3 PR and 3 PS) of *C. lectularius *were categorized into various development stages (early instar nymphs, late instar nymphs and adults) as previously described [19,70]. The tissues (cuticle, midgut and Malphigian tubules) were dissected as per Mamidala et al. [71]. Total RNA was extracted using TriZOL and evaluated with Nanodrop. Further, RNA was treated with TURBO DNase™ (AMBION, Inc., Austin, TX) to remove any genomic contamination, and stored (at -80°C) until further use. First strand cDNA was synthesized in 20 μL reactions using ~0.5 μg of RNA, oligo dT primer and Super Script III First-Strand Synthesis Super Mix (Invitrogen) and the resultant cDNA was diluted to 20 ng/μl for qRT-PCR studies.

### Gene characterization of CYP 397A1V2

To develop full length CYP397A1V2, we performed 3' RACE (Rapid Amplification of cDNA Ends) using an oligo dT primer (Invitrogen) and gene specific primers (Additional file 17). The amplicon was sent for sequencing at the Molecular and Cellular Imaging Center, Wooster, OH, USA. Two additional gene specific primers were designed to the ends of the full length gene to confirm the sequence (Additional file 17). The identification and annotation of the CYP3 clan member was performed using BLASTx search against nr database at NCBI http://www.ncbi.nlm.nih.gov/. For the phylogenetic analysis, an unrooted neighbor-joining tree was constructed with 500 bootstrap replicates and excluding positions with gaps using MEGA version 5 [72].

### Primer designing and qRT-PCR

Twelve potential genes involved in insecticide resistance that showed differential expression in RNA-*Seq *were profiled including 4 cytochrome P450s (CYP9, CYP6A2, CYP6A13 and CYP301A2), 5 cuticular proteins (larval cuticle protein, pupal cuticle protein, cuticle protein analogous to peritrophin, chitin deacetylase and chitin synthase), an antioxidant gene (superoxide dismutase), an acetylcholinesterase gene, and an ABC transporter. Primers were designed using Beacon Designer 7 software (Additional file 17). Prior to qRT-PCR, standard PCR was performed for all primer pairs and their products were run on agarose gel electrophoresis to ensure single bands. qRT-PCR reactions were performed in 96-well plates using a BioRad thermocycler (CFX-96) as per Bai et al. [19]. The analysis included three biological replicates and three technical replicates (within each biological replicate) for RNA-*Seq *validation and for developmental stages whereas two biological replicates (PS1, PS2, PR1 and PR2) and two technical replicates (within each biological replicate) were included for tissues. Relative gene expression among PR and PS adult populations was analyzed as per Bai et al. [19]. For tissues and developmental stages, the fold change in gene expression between PR and PS strains of *C. lectularius *were derived by the 2^-ΔΔCT ^method [73] using ribosomal protein (RPL18) of *C. lectularius *as the internal control gene [71].

### Statistical analysis

The Wilcoxon rank sum test revealed statistically significant differences (*P *< 0.0001) between the adult samples for PR and PS strains [74]. The transformed C_T _values (2^-C^_T_) were used for statistical analysis using SAS (SAS/STAT User's Guide, Version 9.1, SAS Institute Inc.) with a significance level (α) of 0.05. A PROC MIXED analysis of variance (ANOVA) was performed for tissues and developmental stages of PR and PS strains.

### Construction of the CYP397A1V2 model

The MODELLER 9v8 program [75] was used to construct the CYPP397A1V2 structure. MODELLER is a general program that implements comparative protein structure modeling by satisfying spatial restraints in terms of probability density functions. To this end, the crystal structure of Human Cytochrome P450 3A4 (PDB code 1W0E) was used as a 3D template (Additional file 12). The MODELLER program was applied to generate 40 satisfactory models, including the iron-oxo group of CYP397A1V2. The model with the lowest energy and the lowest restraint violation was selected. The initial model was improved by energy minimization. First, energy minimization of 1,000 steps of steepest descent (SD) followed by 2,000 steps of conjugate gradient (CG) was carried out in order to release the conflicting contacts among residues. After performing 2,000 steps of conjugate gradient (CG) minimization, MD simulation was carried out to examine the quality of the model structure, by checking the stability via performing 2000 ps simulations at a constant temperature 300 K. An explicit solvent model SPC216 water was used [76]. All energy minimization and MD simulations were accomplished by GROMACS4.0 software package [77,78] using the GROMOS96 43a1 force field [79,80].

### Molecular docking, preparation of the protein and the ligand

The molecular docking program Autodock, which uses a powerful Lamarkian genetic algorithm (LGA), was used to dock the ligands to the protein active site [81]. Insecticide substrate models (DDT, diazinon, deltamethrin, imidacloprid and permethrin) were built, and their geometry was optimized through discover3 in the InsightII/Builder program http://www.accelrys.com. To recognize the binding sites in CYPP397A1V2, blind docking was carried out with the grid size set to 126, 126 and 126 along the X, Y and Z axes with 0.397 Å grid spacing. The AutoDocking parameters used were GA population size: 150 and maximum number of energy evolutions: 250,000. During docking, a maximum number of 10 conformers was considered, and the root-mean-square (rms) cluster tolerance set to 1.5 Å. One of the lowest energy conformations was considered for further analysis.

### DNA extraction and gene sequencing for *kdr *mutations

For each of the bed bug populations (PS1, PS2, PS3, PR1, PR2, PR3), five whole adults were homogenized and genetic material was extracted using the EZNA DNA extraction kit (Omega Bio-Tek). Gene segments containing known mutation sites in the voltage-gated sodium ion channel were amplified using PCR as described in Zhu et al. [17] (Additional file 17). PCR products were subsequently purified and sequenced by Functional Biosciences (Madison, WI, USA) http://www.functionalbio.com/web/.

### Data deposition

All the contigs and CYP397A1V2 were deposited in Genbank under accession numbers GSE31823 and JN624742, respectively.

## Authors' contributions

PM and OM designed research. PM, AW, SW, BS, LRV and AH performed research. PM, AW, SW, KK, BS and OM contributed tools for data analysis. SCJ provided bed bugs for the study. OM, SCJ, TM and AW contributed reagents and all authors contributed to manuscript preparation and all approved the final manuscript.

## Supplementary Material

Additional file 1 **Transcriptome coverage estimates**. Estimate of transcriptome coverage of ***Cimex lectularius ***using ESTcalc. Click here for file

Additional file 2 **Top BLAST hits**. A pie chart showing distribution of top BLAST hits of *Cimex lectularius *sequences. Click here for file

Additional file 3 **Distribution of *Cimex lectularius *GO categories**. Distribution of GO categories for biological process, cellular component and molecular function. BB-bed bug and DM-*Drosophila melanogaster*. Click here for file

Additional file 4 **Viral proteins in *Cimex lectularius***. The ESTs putatively encoding for viral proteins found in *Cimex lectularius *transcriptome. Click here for file

Additional file 5 **Putative pathways identified in *Cimex lectularius *transcriptome**. KEGG summary of *Cimex lectularius *transcriptome. Click here for file

Additional file 6 **Top 15,000 significant differentials identified in *Cimex lectularius***. The top 15,000 ESTs that differentially expressed among pesticide-resistant and pesticide-susceptible *Cimex lectularius*. Click here for file

Additional file 7 **Gene ontology (GO) term enrichment of top differentials of *Cimex lectularius***. GO term enrichment of top 5,000 differentially expressed ESTs of pesticide-resistant and pesticide-susceptible *Cimex lectularius*. Click here for file

Additional file 8 **Major clades of cytochrome P450 found in *Cimex lectularius *transcriptome**. Classification of various cytochrome P450s in top 5,000 differentially expressed ESTs of *Cimex lectularius *according to their clades. Click here for file

Additional file 9 **Uniquely expressed ESTs**. List of uniquely expressed ESTs among pesticide-resistant and pesticide-exposed strains of *Cimex lectularius*. Click here for file

Additional file 10 **qRT-PCR validation of candidate genes. List of 12 candidate genes selected from top 15,000 differentially expressed ESTs of *C. lectularius *for qRT-PCR validation**. Samples assayed include pesticide-resistant and pesticide-susceptible *C. lectularius*. A *C. lectularius*-specific ribosomal protein (RPL-18) was used as an internal control. Click here for file

Additional file 11 **Characterization of CYP397A1V2 of *Cimex lectularius***. Nucleotide and deduced amino acid sequence of *Cimex lectularius *P450 (*Cl*CYP397A1V2) (A). The first line represents nucleotide sequence and the second line represents amino acid sequence. The amino acids highlighted in grey indicate start codon, signature motifs (helix I;[A/G]GX[E/D]T[T/S], position 297, the helix K motif [EXXRXXP], position 355, a sequence motif [PXXFXP], position 404 and the heme-binding "signature" motif [PFXXGXXXCXG], position 423), stop codon and PolyA tail, respectively. Phylogenetic analysis of *C. lectularius *cytochrome P450 (*Cl*CYP397A1V2) with other cytochrome P450 clan members (B). Letter designation: Ae, *Acromyrmex echinatior*; Aa, *Aedes albopictus*; Ag, *Anopheles gambiae*; Bm, *Bombyx mori*; Cl, *Cimex lectularius*; Cq, *Culex quinquefasciatus*; Hs, *Harpegnathos saltator*; Ms, *Manduca sexta*; Nv, *Nasonia vitripennis*. The topology was derived by unrooted neighbor-joining method with 500 bootstrap replicates using MEGA version 5. The CYP397A1V2 grouped within the CYP6 and CYP9 members of CYP3 clan, leaving the CYP4 clan as an out group. Click here for file

Additional file 12 **Comparison of the deduced amino acid sequence of the *Cimex lectularius *cytochrome P450**. Sequence alignment between CYP397A1V2 of *C. lectularius *and Human Cytochrome P450 CYP3A4 (1WOE). Identity at the amino acid level between the two protein sequences is indicated by the symbol *. Click here for file

Additional file 13 **Ramachandran plot statistics**. Detailed Ramachandran plot statistics for the three dimensional model of CYP397A1V2 of *Cimex lectularius*. Click here for file

Additional file 14 **Molecular docking of CYP397A1V2 with various substrates**. Free energy of Binding, Binding constant (K_a_) and Inhibitory constant (K_i_) for ligands (DDT, imidacloprid, deltamethrin, permethrin and diazinon) docked into the CYP397A1V2 model. Click here for file

Additional file 15 ***kdr *mutations in pesticide-susceptible and pesticide-resistant strains of *Cimex lectularius***. The Valine to Leucine mutation (V419L) and the Leucine to Isoleucine mutation (L925I) was identified in all the three pesticide-resistant strains used in the current study. The two pesticide-susceptible populations (PS1 and PS2) did not carry these mutations. However, the V419L mutation was observed among the PS3 population. Please refer to Zhu et al. [17] who report these mutations in *C. lectularius*. Click here for file

Additional file 16 **Top 30,000 differentials among pesticide-resistant and pesticide-susceptible *Cimex lectularius***. The utility of GLMseq for analysing differential expression among pesticide-resistant and pesticide-susceptible *Cimex lectularius *is better explained. The Contig_18664 clearly suggests a large interaction effect. In this case the GLMseq estimate of the main Resistant/Susceptible fold-change was 1.14, with a two-sided p-value of 0.85, indicating the absence of a significant resistance effect, whereas the Contig_27586 clearly suggests a positive resistance effect. Click here for file

Additional file 17 **Primers used in the current study**. List of oligonucleotide primers used in qRT-PCR validation. Click here for file

## References

[B1] Doggett SL Geary MJ Russel R The resurgence of bedbugs in Australia: with notes on their ecology and control Env Health 2004 4 30 38

[B2] Hwang SW Svoboda TJ De Jong LJ Kabasele KJ Gogosis E Bed bug infestations in an urban environment Emerg Infect Dis 2005 11 533 538 15829190 10.3201/eid1104.041126 PMC3320350

[B3] Harlan JH Bed bug control: challenging and still evolving Outlooks on Pest Management 2007 18 57 61 10.1564/18apr04

[B4] Masetti M Bruschi F Bedbug infestations recorded in central Italy Parasitol Int 2007 56 81 83 10.1016/j.parint.2006.12.002 17258934

[B5] Reinhardt K Harder A Holland S Hooper J Leake-Lyall C Who knows the bed bug? Knowledge of adult bed bug appearance increases with people's age in three counties of Great Britain J Med Entomol 2008 45 956 958 10.1603/0022-2585(2008)45[956:WKTBBK]2.0.CO;2 18826041

[B6] Lee IY Ree HI An SJ Linton JA Yong TS Reemergence of the bedbug *Cimex lectularius *in Seoul, Korea Korean J Parasitol 2008 46 269 271 10.3347/kjp.2008.46.4.269 19127335 PMC2612614

[B7] Delaunay P Blanc V Del Giudice P Levy-Bencheton A Chosidow O Marty P Brouqui P Bedbugs and infectious diseases Clin Infect Dis 2011 52 200 210 10.1093/cid/ciq102 21288844 PMC3060893

[B8] CDC 2011 http://www.cdc.gov/parasites/bedbugs/health_professionals/index.html

[B9] Goddard J de Shazo RD Bedbugs (*Cimex lectularius*) and clinical consequences of their bites JAMA 2009 301 1358 1366 10.1001/jama.2009.405 19336711

[B10] Lowe CF Romney MG Bedbugs as vectors for drug-resistant bacteria Emer Infect Dis 2011 17 1132 1134 10.3201/eid1706.101978 PMC3358218 21749792

[B11] Busvine JR Insecticide-resistant strains of insects of public health importance Trans R Soc Trop Med Hyg 1957 51 11 31 10.1016/0035-9203(57)90002-0 13409589

[B12] Lofgren CS Keller JC Burden GS Resistance tests with the bed bug and evaluation of insecticides for its control J Econ Entomol 1958 51 241 244

[B13] Romero A Potter MF Potter DA Haynes KF Insecticide resistance in the bed bug: a factor in the pest's sudden resurgence? J Med Entomol 2007 44 175 178 10.1603/0022-2585(2007)44[175:IRITBB]2.0.CO;2 17427684

[B14] Boase C Takken W, Knols BGJ Bed bugs: research and resurgence Emerging pests and vector-borne diseases in Europe 2007 1 Wageningen Academic Publishers, Wageningen 261 280

[B15] Anderson A The decade of bedbugs and fear Environ Health Insights 2011 5 53 54 21695091 10.4137/EHI.S6923 PMC3115641

[B16] Mamidala P Jones SC Mittapalli O Metabolic resistance in bed bugs Insects 2011 2 36 48 10.3390/insects2010036 PMC4553422 26467498

[B17] Zhu F Wigginton J Romero A Moore A Ferguson K Palli R Potter MF Haynes KF Palli SR Widespread distribution of knockdown resistance mutations in the bed bug, *Cimex lectularius *(Hemiptera: Cimicidae), populations in the United States Arch Insect Biochem Physiol 2010 73 245 257 20301216 10.1002/arch.20355

[B18] Yoon KS Kwon DH Strycharz JP Hollingsworth CS Lee SH Clark JM Biochemical and molecular analysis of deltamethrin resistance in the common bed bug (Hemiptera: Cimicidae) J Med Entomol 2008 45 1092 1101 10.1603/0022-2585(2008)45[1092:BAMAOD]2.0.CO;2 19058634

[B19] Bai X Mamidala P Rajarapu SP Jones SC Mittapalli O Transcriptomics of the bed bug (*Cimex lectularius*) PLoS ONE 2011 6 e16336 10.1371/journal.pone.0016336 21283830 PMC3023805

[B20] Adelman ZN Kilcullen KA Koganemaru R Anderson MAE Anderson TD Miller DM Deep sequencing of pyrethroid-resistant bed bugs reveals multiple mechanisms of resistance within a single population PLoS ONE 2011 6 e26228 10.1371/journal.pone.0026228 22039447 PMC3198472

[B21] Li P Ponnala L Gandotra N Wang L Si Y Tausta SL Kebrom TH Provart N Patel R Myers CR The developmental dynamics of the maize leaf transcriptome Nat Genet 2010 42 1060 1067 10.1038/ng.703 21037569

[B22] Wang Z Gerstein M Snyder M RNA-Seq: a revolutionary tool for transcriptomics Nat Rev Genet 2009 10 57 63 10.1038/nrg2484 19015660 PMC2949280

[B23] Siebert S Robinson MD Tintori SC Goetz F Helm RR Smith SA Shaner N Haddock SH Dunn CW Differential gene expression in the siphonophore *Nanomia bijuga *(Cnidaria) assessed with multiple next-generation sequencing workflows PLoS ONE 2011 6 e22953 10.1371/journal.pone.0022953 21829563 PMC3146525

[B24] Crawford JE Guelbeogo WM Sanou A Traoré A Vernick KD Sagnon N Lazzaro BP *De Novo *Transcriptome Sequencing in *Anopheles funestus *Using Illumina RNA-Seq technology PLoS ONE 2010 5 e14202 10.1371/journal.pone.0014202 21151993 PMC2996306

[B25] Bonizzoni M Dunn WA Campbell CL Olson KE Dimon MT Marinotti O James AA RNA-seq analyses of blood-induced changes in gene expression in the mosquito vector species, *Aedes aegypti* BMC Genomics 2011 12 82 10.1186/1471-2164-12-82 21276245 PMC3042412

[B26] Liu F Li W Li Z Zhang S Chen S Su S High-abundance mRNAs in *Apis mellifera*: Comparison between nurses and foragers J Insect Physiol 2011 57 274 279 10.1016/j.jinsphys.2010.11.015 21115016

[B27] Pitts RJ Rinker DC Jones PL Rokas A Zwiebel LJ Transcriptome profiling of chemosensory appendages in the malaria vector *Anopheles gambiae *reveals tissue- and sex-specific signatures of odor coding BMC Genomics 2011 27 271 10.1186/1471-2164-12-271 PMC3126782 21619637

[B28] DiGuistini S Wang Y Liao NY Taylor G Tanguay P Feau N Henrissat B Chan SK Hesse-Orce U Alamouti SM Genome and transcriptome analyses of the mountain pine beetle-fungal symbiont *Grosmannia clavigera*, a lodgepole pine pathogen Proc Natl Acad Sci USA 2011 108 2504 2509 10.1073/pnas.1011289108 21262841 PMC3038703

[B29] O'Neil ST Dzurisin JD Carmichael RD Lobo NF Emrich SJ Hellmann JJ Population-level transcriptome sequencing of nonmodel organisms *Erynnis propertius *and *Papilio zelicaon* BMC Genomics 2010 11 310 10.1186/1471-2164-11-310 20478048 PMC2887415

[B30] Pauchet Y Wilkinson P van Munster M Augustin S Pauron D Ffrench-Constant RH Pyrosequencing of the midgut transcriptome of the poplar leaf beetle *Chrysomela tremulae *reveals new gene families in Coleoptera Insect Biochem Mol Biol 2009 39 403 13 10.1016/j.ibmb.2009.04.001 19364528

[B31] Karatolos N Pauchet Y Wilkinson P Chauhan R Denholm I Gorman K Nelson DR Bass C Pyrosequencing the transcriptome of the greenhouse whitefly, *Trialeurodes vaporariorum *reveals multiple transcripts encoding insecticide targets and detoxifying enzymes BMC Genomics 2011 12 56 10.1186/1471-2164-12-56 21261962 PMC3036619

[B32] Pedra JHF McIntyre LM Scharf ME Pittendrigh BR Genome-wide transcription profile of field- and laboratory-selected dichlorodiphenyltrichloroethane (DDT)-resistant *Drosophila* Proc Natl Acad Sci USA 2004 101 7034 7039 10.1073/pnas.0400580101 15118106 PMC406461

[B33] Liu N Liu H Zhu F Zhang L Differential expression of genes in pyrethroid resistant and susceptible mosquitoes, *Culex quinquefasciatus *(S.) Gene 2007 394 61 68 10.1016/j.gene.2007.01.032 17382491

[B34] Labbe R Caveney S Donly C Genetic analysis of the xenobiotic resistance associated *ABC *gene subfamilies of the Lepidoptera Insect Mol Biol 2011 20 243 256 10.1111/j.1365-2583.2010.01064.x 21199020

[B35] Mittapalli O Neal JJ Shukle RH Antioxidant defense response in a galling insect Proc Natl Acad Sci USA 2007 104 1889 1894 10.1073/pnas.0604722104 17261812 PMC1783901

[B36] Scott JG Cytochromes P450 and insecticide resistance Insect Biochem Mol Biol 1999 29 757 777 10.1016/S0965-1748(99)00038-7 10510498

[B37] Strode C Wondji CS David JP Hawkes NJ Lumjuan N Nelson DR Drane DR Karunaratne SH Hemingway J Black WC Ranson H Genomic analysis of detoxification genes in the mosquito *Aedes aegypti* Insect Biochem Mol Biol 2008 38 113 123 10.1016/j.ibmb.2007.09.007 18070670

[B38] Brun-Barale A Héma O Martin T Suraporn S Audant P Sezutsu H Feyereisen R Multiple P450 genes overexpressed in deltamethrin-resistant strains of *Helicoverpa armigera* Pest Manag Sci 2010 66 900 909 20540120 10.1002/ps.1960

[B39] Awolola TS Oduola OA Strode C Koekemoer LL Brooke B Ranson H Evidence of multiple pyrethroid resistance mechanisms in the malaria vector *Anopheles gambiae sensu stricto *from Nigeria Trans R Soc Trop Med Hyg 2009 103 1139 1145 10.1016/j.trstmh.2008.08.021 18829056

[B40] Brandt A Scharf M Pedra JHF Holmes G Dean A Kreitman M Pittendrigh BR Differential expression and induction of two *Drosophila *cytochrome P450 genes near the Rst (2) DDT locus Insect Mol Biol 2002 11 337 341 10.1046/j.1365-2583.2002.00344.x 12144699

[B41] Feyereisen R Evolution of insect P450 Biochem Soc Trans 2006 34 1252 1255 17073796 10.1042/BST0341252

[B42] Mittapalli O Bai X Mamidala P Rajarapu SP Bonello P Herms DA Tissue specific transcriptomics of the exotic invasive insect pest emerald ash borer PLoS ONE 2010 5 e13708 10.1371/journal.pone.0013708 21060843 PMC2965670

[B43] Bogwitz MR Chung H Magoc L Rigby S Wong W O'Keefe M McKenzie JA Batterham P CYP12A4 confers lufenuron resistance in a natural population of *Drosophila melanogaster* Proc Natl Acad Sci USA 2005 102 12807 12812 10.1073/pnas.0503709102 16120680 PMC1200273

[B44] Guzov VM Unnithan GC Chernogolov AA Feyereisen R CYP12A1, a mitochondrial cytochrome P450 from the house fly Arch Biochem Biophys 1998 359 231 240 10.1006/abbi.1998.0901 9808765

[B45] Rewitz KF Rybczynski R Warren JT Gilbert LI The Halloween genes code for cytochrome P450 enzymes mediating synthesis of the insect moulting hormone Biochem Soc Trans 2006 34 1256 1260 10.1042/BST0341256 17073797

[B46] Rewitz KF Gilbert LI Daphnia Halloween genes that encode cytochrome P450s mediating the synthesis of the arthropod molting hormone: evolutionary implications BMC Evol Biol 2008 8 60 10.1186/1471-2148-8-60 18298845 PMC2276477

[B47] Wood OR Hanrahan S Cuticle thickening associated with pyrethroid resistance in the major malaria vector *Anopheles funestus* Parasites and Vectors 2010 3 67 10.1186/1756-3305-3-67 20684757 PMC2924294

[B48] Wigglesworth B The physiology of insect cuticle Ann Rev Entomol 1957 2 37 54 10.1146/annurev.en.02.010157.000345

[B49] Arakane Y Dixit R Begum K Park Y Specht CA Merzendorfer H Kramer KJ Muthukrishnan S Beeman RW Analysis of functions of the chitin deacetylase gene family in *Tribolium castaneum* Insect Biochem Mol Biol 2009 39 355 365 10.1016/j.ibmb.2009.02.002 19268706

[B50] Abedi ZH Brown AWA Peritrophic membrane as vehicle for DDT and DDE excretion in *Aedes aegypti *larvae Ann Entomol Soc Am 1961 54 539 542

[B51] Elvin CM Vuocolo T Pearson RD East IJ Riding GA Eisemann CH Tellam RL Characterization of a major peritrophic membrane protein, Peritrophin-44, from the larvae of *Lucilia cuprina *cDNA and deduced amino acid sequences J Biol Chem 1996 271 8925 35 10.1074/jbc.271.15.8925 8621536

[B52] Vontas J Blass C Koutsos AC David JP Kafatos FC Louis C Hemingway J Christophides GK Ranson H Gene expression in insecticide resistant and susceptible *Anopheles gambiae *strains constitutively or after insecticide exposure Insect Mol Biol 2005 14 509 521 10.1111/j.1365-2583.2005.00582.x 16164607

[B53] Labbe R Caveney S Donly C Genetic analysis of the xenobiotic resistance-associated *ABC *gene subfamilies of the Lepidoptera Insect Mol Biol 2010 20 243 256 21199020 10.1111/j.1365-2583.2010.01064.x

[B54] Revuelta L Piulachs MD Bellés X Castañera P Ortego F Díaz-Ruíz JR Hernández-Crespo P Tenllado F RNAi of ace1 and ace2 in *Blattella germanica *reveals their differential contribution to acetylcholinesterase activity and sensitivity to insecticides Insect Biochem Mol Biol 2009 39 913 919 10.1016/j.ibmb.2009.11.001 19900550

[B55] Chung H Sztal T Pasricha S Sridhar M Batterham P Daborn PJ Characterization of *Drosophila melanogaster *cytochrome P450 genes Proc Natl Acad Sci USA 2009 106 5731 5736 10.1073/pnas.0812141106 19289821 PMC2667016

[B56] Aw T Schlauch K Keeling CI Young S Bearfield JC Blomquist GJ Tittiger C Functional genomics of mountain pine beetle (*Dendroctonus ponderosae*) midguts and fat bodies BMC Genomics 2010 11 215 20353591 10.1186/1471-2164-11-215 PMC2858752

[B57] Zhu F Parthasarathy R Bai H Woithe K Kaussmann M Nauen R Harrison DA Palli SR A brain-specific cytochrome P450 responsible for the majority of deltamethrin resistance in the QTC279 strain of *Tribolium castaneum* Proc Natl Acad Sci USA 2010 107 8557 8562 10.1073/pnas.1000059107 20410462 PMC2889294

[B58] Zhou X Ma C Li M Sheng C Liu H Qiu X *CYP9A12 *and *CYP9A17 *in the cotton bollworm, *Helicoverpa armigera*: sequence similarity, expression profile and xenobiotic response Pest Manag Sci 2010 66 65 73 10.1002/ps.1832 19728321

[B59] Davies TGE Field LM Usherwood PNR Williamson MS DDT, pyrethrins, pyrethroids, and insect sodium channels IUBMB Life 2007 59 151 162 10.1080/15216540701352042 17487686

[B60] Romero A Potter MF Haynes KF Insecticide resistant bed bugs: implications for the industry Pest Control Technol 2007 35 42 50

[B61] Li W Schuler MA Berenbaum MR Diversification of furanocoumarin metabolizing cytochrome P450 monooxygenases in two papilionids: specificity and substrate encounter rate Proc Natl Acad Sci USA 2003 100 14593 14598 10.1073/pnas.1934643100 12968082 PMC304124

[B62] Martin J Bruno VM Fang Z Meng X Blow M Zhang T Sherlock G Snyder M Wang Z Rnnotator: an automated *de novo *transcriptome assembly pipeline from stranded RNA-Seq reads BMC Genomics 2010 11 663 10.1186/1471-2164-11-663 21106091 PMC3152782

[B63] Sommer DD Delcher AL Salzberg SL Pop M Minimus: a fast, lightweight genome assembler BMC Bioinformatics 2007 8 64 10.1186/1471-2105-8-64 17324286 PMC1821043

[B64] Ewen-Campen B Shaner N Panfilio KA Suzuki Y Roth S Extavour CG The maternal and early embryonic transcriptome of the milkweed bug *Oncopeltus fasciatus* BMC Genomics 2011 12 61 10.1186/1471-2164-12-61 21266083 PMC3040728

[B65] Conesa A Götz S García-Gómez JM Terol J Talón M Robles M Blast2GO: a universal tool for annotation, visualization and analysis in functional genomics research Bioinformatics 2005 21 3674 3676 10.1093/bioinformatics/bti610 16081474

[B66] Ogata H Goto S Sato K Fujibuchi W Bono H Kanehisa M KEGG: Kyoto Encyclopedia of Genes and Genomes Nucleic Acids Res 1999 27 29 34 10.1093/nar/27.1.29 9847135 PMC148090

[B67] B2G-FAR: A Species Centered GO Annotation Repository. http://bioinfo.cipf.es/b2gfar/showspecies?species=7227 10.1093/bioinformatics/btr059 PMC3065692 21335611

[B68] Hu Z Jie B Reecy J CateGOrizer: A Web-Based Program to Batch Analyze Gene Ontology Classification Categories Online Journal of Bioinform 2008 9 108 112

[B69] Risso D Schwartz K Sherlock G Dudoit S GC-Content Normalization for RNA-Seq Data 2011 University of California, Berkeley, Division of Biostatistics http://www.bepress.com/ucbbiostat/paper291/Technical report #291 10.1186/1471-2105-12-480 PMC3315510 22177264

[B70] Usinger R Monograph of Cimicidae. Thomas Say Foundation, Vol. 7. College Park, MD: Entomological Society of America 1966

[B71] Mamidala P Rajarapu SP Jones SC Mittapalli O Identification and validation of reference genes for quantitative real-time PCR in the bed bug J Med Entomol 2011 48 947 951 10.1603/ME10262 21845960

[B72] Tamura K Peterson D Peterson N Stecher G Nei M Kumar S MEGA5: Molecular evolutionary genetics analysis using maximum likelihood, evolutionary distance, and maximum parsimony methods Mol Biol Evol 2011 in press doi:10.1093/molbev/msr121 10.1093/molbev/msr121 PMC3203626 21546353

[B73] Schmittgen TD Livak KJ Analyzing real-time PCR data by the comparative C(T) method Nat Protocols 2008 3 1101 1118 10.1038/nprot.2008.73 18546601

[B74] R Development Core Team R: A language and environment for statistical computing R Foundation for Statistical Computing 2010 Vienna, Austria ISBN 3-900051-07-0, URL http://www.R-project.org

[B75] Sali A Blundell TL Comparative protein modeling by satisfaction of spatial restraints J Mol Biol 1993 234 779 815 10.1006/jmbi.1993.1626 8254673

[B76] Berendsen HJC Postma JPM van Gunsteren WF Hermans J Pullman B Interaction models for water in relation to protein hydration Intermolecular forces 1981 331 342

[B77] Berendsen HJC van der Spoel D van Drunen R GROMACS: a message-passing parallel molecular dynamics implementation Comp Phys Comm 1995 91 43 56 10.1016/0010-4655(95)00042-E

[B78] Lindah E Hess B van der Spoel D GROMACS 3.0: a package for molecular simulation and trajectory analysis J Mol Model 2001 7 306 317

[B79] Van Gunsteren WF Billeter SR Eising AA Hünenberger PH Krüger PKHC Mark AE Scott WRP Tironi IG Biomolecular simulation: The GROMOS96 manual and user guide 1996 Vdf Hochschulverlag AG, Zürich

[B80] Van Gunsteren WF Daura X Mark AE Von Rague Schleyer P The GROMOS force field Encycloped Computat Chem 1998 1211 1216

[B81] Morris GM Goodsell DS Halliday RS Huey R Hart WE Belew RK Olson AJ Automated docking using a Lamarckian genetic algorithm and an empirical binding free energy function J Comput Chem 1998 19 1639 1662 10.1002/(SICI)1096-987X(19981115)19:14<1639::AID-JCC10>3.0.CO;2-B

