# Supplementary material for: RNA-Seq and molecular docking reveal multi-level pesticide resistance in the bed bug
Source: BMC Genomics. 2012 Jan 6;13:6. doi: 10.1186/1471-2164-13-6 (PMC3273426; doi:10.1186/1471-2164-13-6)
Supplement: Additional file 1 — Transcriptome coverage estimates. Estimate of transcriptome coverage of Cimex lectularius using ESTcalc. [file 1471-2164-13-6-S1.DOC]

### Additional file 1. Transcriptome coverage estimates.

|  | **Predicted** | **Actual** |
| --- | --- | --- |
| Number of technologies | 2 |  |
| Technology 1 | 454 GSFLX |  |
| Library type | non-normalized |  |
| Number of Megabases | 100 | 71.5 |
| Technology 2 | Solexa |  |
| Library type | non-normalized |  |
| Number of Megabases | 4000 | 5068 |
|  |  |  |
|  | Output |  |
| Total Sequence Amount (MB) | 4100 | 5139.5 |
| Total Assembled Sequence (MB) | 31.4 | 33.8 |
| Unigene count | 111192 | 51,492 |
| Mean unigene length (bp) | 282 | 1150 |
| Mean unigene length (longest unigene per gene, bp) | 1329 |  |
| Singleton yield (%) | 7 |  |
| Percent transcriptome (%) | 100 |  |
| Percent of genes tagged (%) | 100 |  |
| Percent of genes with 90% coverage (%) | 100 |  |
| Percent of genes with 90% coverage by largest unigene (%) | 77.6 |  |
| Percent of genes with 100% coverage (%) | 98.7 |  |
