# Supplementary figures and images for: RNA-Seq and molecular docking reveal multi-level pesticide resistance in the bed bug
Source: BMC Genomics. 2012 Jan 6;13:6. doi: 10.1186/1471-2164-13-6 (PMC3273426; doi:10.1186/1471-2164-13-6)

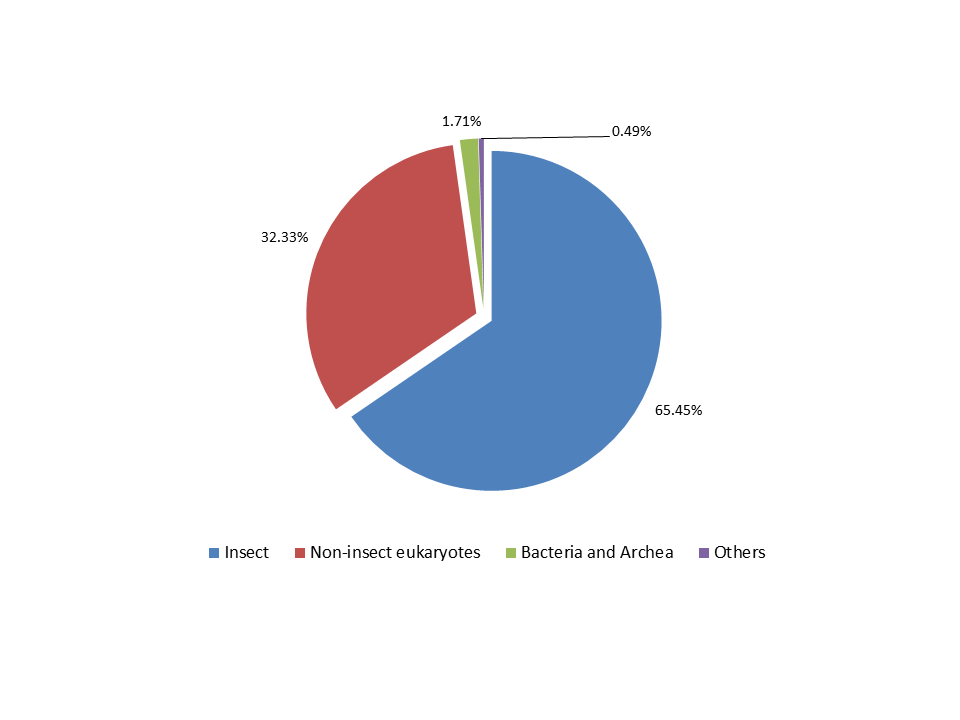

Supplement: Additional file 2 — Top BLAST hits. A pie chart showing distribution of top BLAST hits of Cimex lectularius sequences. [file 1471-2164-13-6-S2.TIFF]

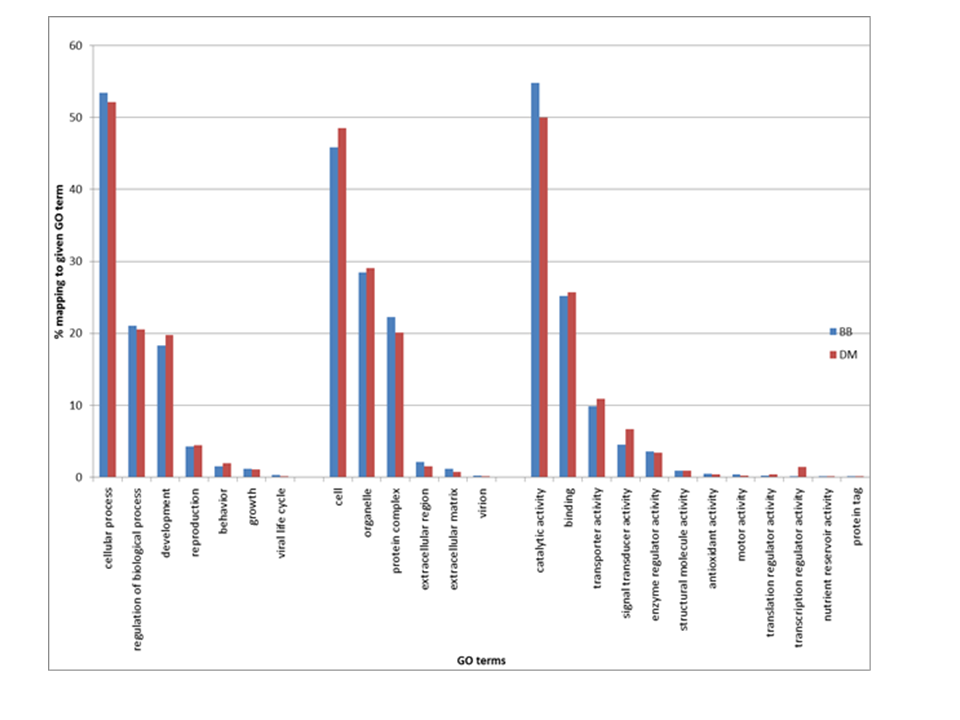

Supplement: Additional file 3 — Distribution of Cimex lectularius GO categories. Distribution of GO categories for biological process, cellular component and molecular function. BB-bed bug and DM-Drosophila melanogaster. [file 1471-2164-13-6-S3.TIFF]

**Additional File 11: Characterization of CYP397A1V2 of *Cimex lectularius***

A.


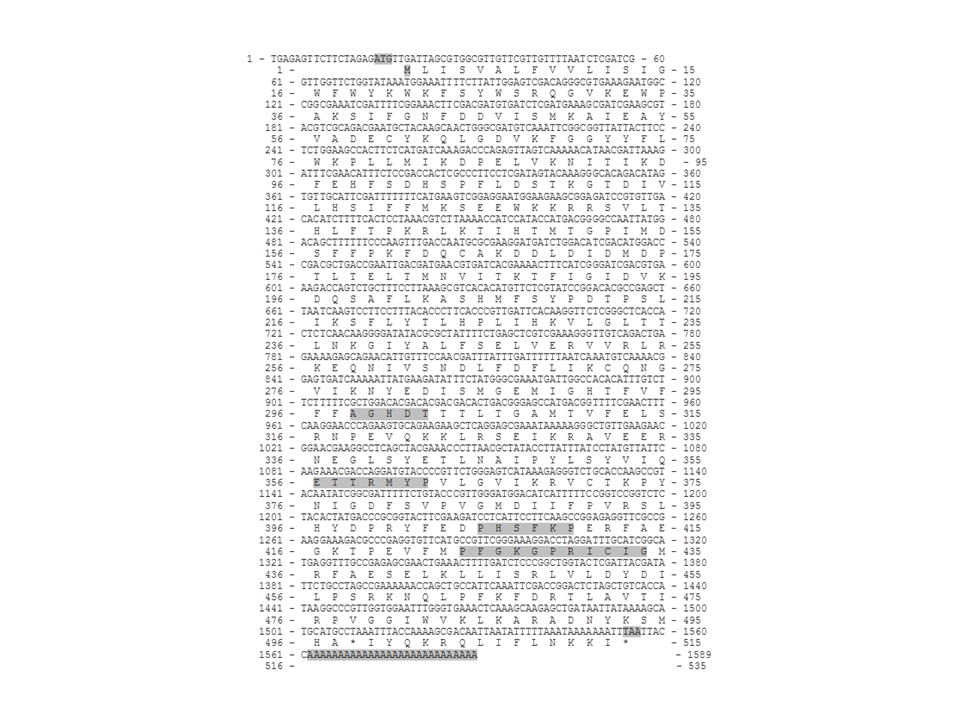


B.


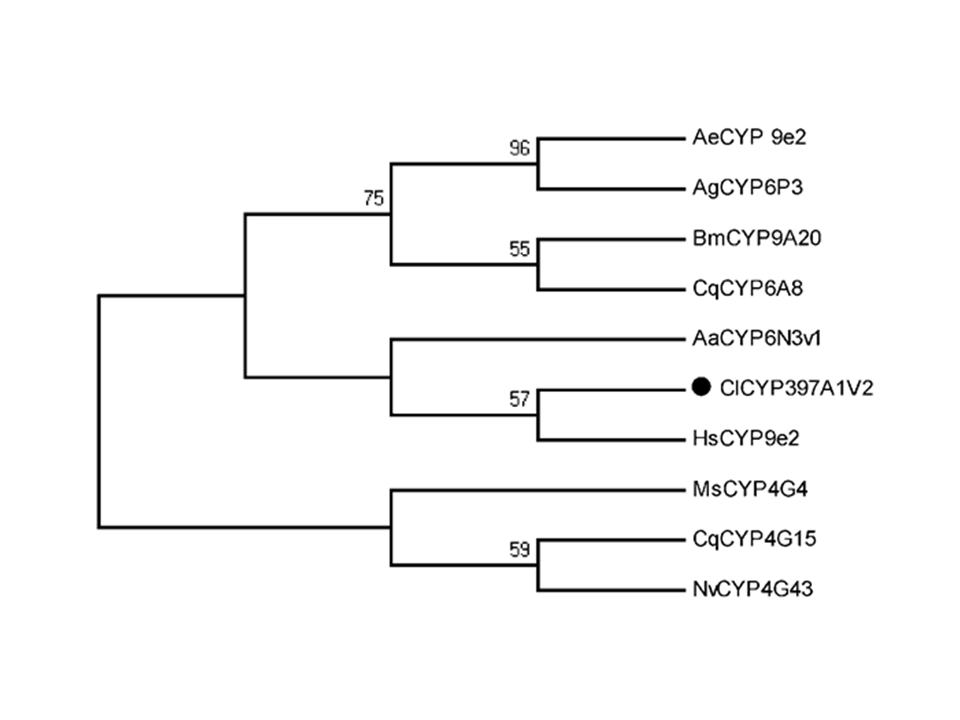

Supplement: Additional file 11 — Characterization of CYP397A1V2 of Cimex lectularius. Nucleotide and deduced amino acid sequence of Cimex lectularius P450 (ClCYP397A1V2) (A). The first line represents nucleotide sequence and the second line represents amino acid sequence. The amino acids highlighted in grey indicate start codon, signature motifs (helix I;[A/G]GX[E/D]T[T/S], position 297, the helix K motif [EXXRXXP], position 355, a sequence motif [PXXFXP], position 404 and the heme-binding "signature" motif [PFXXGXXXCXG], position 423), stop codon and PolyA tail, respectively. Phylogenetic analysis of C. lectularius cytochrome P450 (ClCYP397A1V2) with other cytochrome P450 clan members (B). Letter designation: Ae, Acromyrmex echinatior; Aa, Aedes albopictus; Ag, Anopheles gambiae; Bm, Bombyx mori; Cl, Cimex lectularius; Cq, Culex quinquefasciatus; Hs, Harpegnathos saltator; Ms, Manduca sexta; Nv, Nasonia vitripennis. The topology was derived by unrooted neighbor-joining method with 500 bootstrap replicates using MEGA version 5. The CYP397A1V2 grouped within the CYP6 and CYP9 members of CYP3 clan, leaving the CYP4 clan as an out group. [file 1471-2164-13-6-S11.DOC]

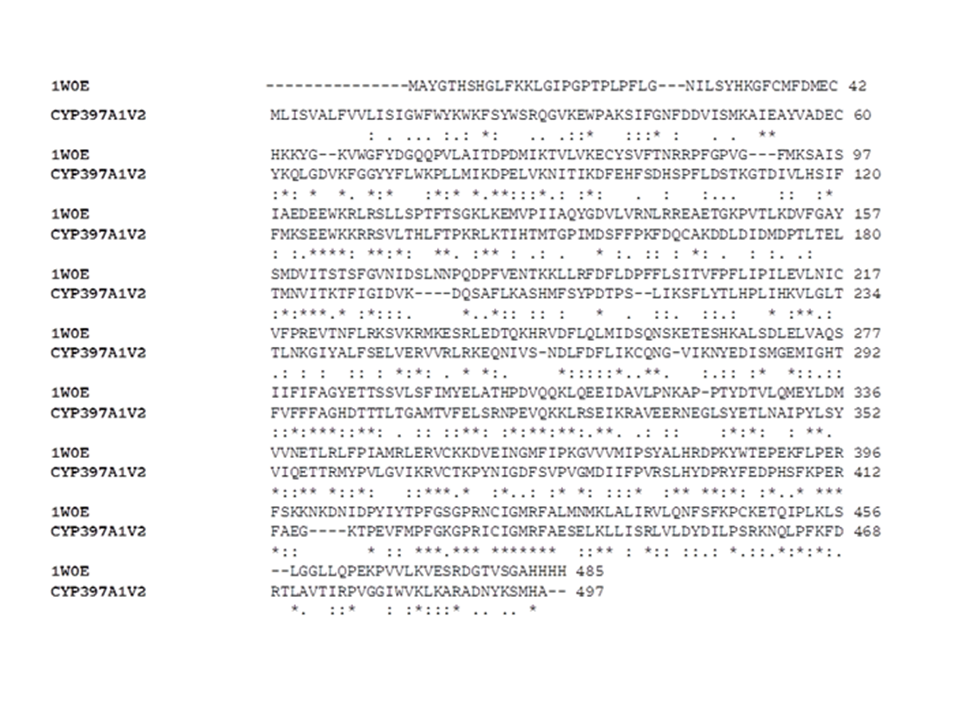

Supplement: Additional file 12 — Comparison of the deduced amino acid sequence of the Cimex lectularius cytochrome P450. Sequence alignment between CYP397A1V2 of C. lectularius and Human Cytochrome P450 CYP3A4 (1WOE). Identity at the amino acid level between the two protein sequences is indicated by the symbol *. [file 1471-2164-13-6-S12.TIFF]
