# Supplementary material for: RNA-Seq and molecular docking reveal multi-level pesticide resistance in the bed bug
Source: BMC Genomics. 2012 Jan 6;13:6. doi: 10.1186/1471-2164-13-6 (PMC3273426; doi:10.1186/1471-2164-13-6)
Supplement: Additional file 10 — qRT-PCR validation of candidate genes. List of 12 candidate genes selected from top 15,000 differentially expressed ESTs of C. lectularius for qRT-PCR validation. Samples assayed include pesticide-resistant and pesticide-susceptible C. lectularius. A C. lectularius-specific ribosomal protein (RPL-18) was used as an internal control. [file 1471-2164-13-6-S10.DOC]

***Additional file 10****: qRT-PCR validation of candidate genes.*

| Contig Number   | Name of the gene | Susceptible  Mean AEV  (s.d., range) | Resistant  Mean AEV  (s.d., range) | W | *P* value |  | | --- | --- | --- | --- | --- | --- | | 1766 | Chitin deacteylase | 0.74 | 3.90 | 0 | < 0.0001 * | | (0.49, 0.26-1.57) | (2.23, 2.04-7.03) |  |  | | 3653 | Acetylcholinesterase | 0.49 | 2.06 | 8 | 0.0027* | | (0.26, 0.22-0.92) | (1.78, 0.50-4.83) |  |  | | 19601 | CYP397A1V2 | 0.22 | 1.39 | 12 | 0.0106 * | | (0.21, 0.01-0.58) | (0.82, 0.07-2.27) |  |  | | 103 | CYP6A2 | 0.08 | 2.65 | 0 | < 0.0001 * | | (0.02, 0.05-0.11) | (3.31, 0.36-7.72) |  |  | | 22399 | CYP6A13 | 0.24 | 1.55 | 12 | 0.0106 * | | (0.22, 0.01-0.62) | (0.93, 0.06-2.48) |  |  | | 1762 | Chitin synthase | 0.34 | 2.23 | 0 | < 0.0001 * | | (0.15, 0.16-0.62) | (1.95, 0.72-5.43) |  |  | | 48951 | CPAP | 0.50 | 2.85 | 4 | 0.0005 * | | (0.27, 0.16-0.95) | (3.51, 0.75-11.4) |  |  | | 18015 | CYP301A2 | 0.02 | 0.02 | 27 | 0.0258 n.s. | | (0.01, 0.01-0.04) | (0.01, 0.02-0.03) |  |  | | 21630 | Pupal cuticle protein | 0.62 | 4.96 | 0 | < 0.0001 * | | (0.50, 0.12-1.38) | (3.78, 1.4-10.7) |  |  | | 17694 | Larval cuticle protein | 0.10 | 0.50 | 7 | 0.0018 * | | (0.06, 0.05-0.26) | (0.36, 0.11-1.21) |  |  | | 49102 | Superoxide dismutase | 0.38 | 2.24 | 0 | < 0.0001 * | | (0.05, 0.31-0.45) | (1.74, 0.82-4.77) |  |  | | 1346 | ABC transporter | 0.55 | 2.12 | 0 | < 0.0001 * | | (0.11, 0.40-0.70) | (1.68, 0.72-4.46) |  |  | |
| --- | --- | --- | --- | --- | --- | --- | --- | --- | --- | --- | --- | --- | --- | --- | --- | --- | --- | --- | --- | --- | --- | --- | --- | --- | --- | --- | --- | --- | --- | --- | --- | --- | --- | --- | --- | --- | --- | --- | --- | --- | --- | --- | --- | --- | --- | --- | --- | --- | --- | --- | --- | --- | --- | --- | --- | --- | --- | --- | --- | --- | --- | --- | --- | --- | --- | --- | --- | --- | --- | --- | --- | --- | --- | --- | --- | --- | --- | --- | --- | --- | --- | --- | --- | --- | --- | --- | --- | --- | --- | --- | --- | --- | --- | --- | --- | --- | --- | --- | --- | --- | --- | --- | --- | --- | --- | --- | --- | --- | --- | --- | --- | --- | --- | --- | --- | --- | --- | --- | --- | --- | --- | --- | --- | --- | --- | --- |
| s.d. = standard deviation, n.s. = not significant, * = statistically significant difference at α = 0.05 as calculated by the Wilcoxon rank sum test. |
