# Supplementary material for: RNA-Seq and molecular docking reveal multi-level pesticide resistance in the bed bug
Source: BMC Genomics. 2012 Jan 6;13:6. doi: 10.1186/1471-2164-13-6 (PMC3273426; doi:10.1186/1471-2164-13-6)
Supplement: Additional file 13 — Ramachandran plot statistics. Detailed Ramachandran plot statistics for the three dimensional model of CYP397A1V2 of Cimex lectularius. [file 1471-2164-13-6-S13.DOC]

**Additional file 13:** Ramachandran plot statistics

______________________________________________________________________________

Sequence Generously

identity Core Allowed allowed Disallowed

______________________________________________________________________________

CYP397A1V2 28% 76.1% 18.2% 4.1% 1.7%

______________________________________________________________________________
