# Supplementary material for: RNA-Seq and molecular docking reveal multi-level pesticide resistance in the bed bug
Source: BMC Genomics. 2012 Jan 6;13:6. doi: 10.1186/1471-2164-13-6 (PMC3273426; doi:10.1186/1471-2164-13-6)
Supplement: Additional file 14 — Molecular docking of CYP397A1V2 with various substrates. Free energy of Binding, Binding constant (Ka) and Inhibitory constant (Ki) for ligands (DDT, imidacloprid, deltamethrin, permethrin and diazinon) docked into the CYP397A1V2 model. [file 1471-2164-13-6-S14.DOC]

**Additional file 14.** Molecular docking of CYP397A1V2 with various substrates.

**______________________________________________________________________________**

**Binding Energy Ligand efficiency**

**Compound (Kcal M^-1^) K_i_  K_a_ (M^-1^)**

**______________________________________________________________________________**

DDT -8.84 330.51 nM 3.02 x 10^6^ -0.47

Imidacloprid -6.93 8.32 μM 1.20 x 10^5^  -0.41

Deltamethrin -11.12 7.11 nM 1.40 x 10^8^ -0.40

Permethrin-10.16 35.71 nM 2.80 x 10^7^  -0.39

Diazinon -6.68 12.66 μM 7.90 x 10^3^ -0.35
