# Supplementary material for: RNA-Seq and molecular docking reveal multi-level pesticide resistance in the bed bug
Source: BMC Genomics. 2012 Jan 6;13:6. doi: 10.1186/1471-2164-13-6 (PMC3273426; doi:10.1186/1471-2164-13-6)
Supplement: Additional file 15 — kdr mutations in pesticide-susceptible and pesticide-resistant strains of Cimex lectularius. The Valine to Leucine mutation (V419L) and the Leucine to Isoleucine mutation (L925I) was identified in all the three pesticide-resistant strains used in the current study. The two pesticide-susceptible populations (PS1 and PS2) did not carry these mutations. However, the V419L mutation was observed among the PS3 population. Please refer to Zhu et al. [17] who report these mutations in C. lectularius. [file 1471-2164-13-6-S15.DOC]

**Additional file 15.** *kdr* mutations in pesticide-susceptible and pesticide-resistant strains of *Cimex lectularius.*

| **Population** | **V419L** | **L925I** |
| --- | --- | --- |
| Harlan1 (PS1) | SAGPWHMLFFIVIIFLGSFYL**V**NLILAIVA | LAKSWPTLNLLISIMGRTVGA**L**GNLTFVLC |
| Harlan2  (PS2) | SAGPWHMLFFIVIIFLGSFYL**V**NLILAIVA | LAKSWPTLNLLISIMGRTVGA**L**GNLTFVLC |
| FV  (PS3) | SAGPWHMLFFIVIIFLGSFYL**L**NLILAIVA | LAKSWPTLNLLISIMGRTVGA**L**GNLTFVLC |
| (PR1) | SAGPWHMLFFIVIIFLGSFYL**L**NLILAIVA | LAKSWPTLNLLISIMGRTVGA**I**GNLTFVLC |
| (PR2) | SAGPWHMLFFIVIIFLGSFYL**L**NLILAIVA | LAKSWPTLNLLISIMGRTVGA**I**GNLTFVLC |
| (PR3) | SAGPWHMLFFIVIIFLGSFYL**L**NLILAIVA | LAKSWPTLNLLISIMGRTVGA**I**GNLTFVLC |
