# Supplementary material for: RNA-Seq and molecular docking reveal multi-level pesticide resistance in the bed bug
Source: BMC Genomics. 2012 Jan 6;13:6. doi: 10.1186/1471-2164-13-6 (PMC3273426; doi:10.1186/1471-2164-13-6)
Supplement: Additional file 17 — Primers used in the current study. List of oligonucleotide primers used in qRT-PCR validation. [file 1471-2164-13-6-S17.DOC]

**Additional file 17**. Primers used in the current study.

| Name of the gene | Forward primer (5’-3’) | Reverse primer (5’-3’) |
| --- | --- | --- |
| *Primers used for quantitative real time PCR* | | |
| BB_Contig_19601 | TCGGAGGAATGGAAGAAG | CGTCATGGTATGGATGGT |
| BB_Contig_103 | AAGTTGTCCTAGAGTGTT | GAGATATGCGTGAATGTC |
| BB_Contig_18015 | CTAAGCCGCATAAGAAGCATCA | CAGTGGAACAGAACCGAAGAC |
| BB_Contig_22399 | CGTCATGGTATGGATGGT | TCGGAGGAATGGAAGAAG |
| BB_Contig_3653 | GACACTAACAACCACCACAT | ACGATTCTACTACTTAACAACCTT |
| BB_Contig_1346 | TGCTCTACATAATTCTGACAT | GTAGGACGGTATGAGGTA |
| BB_Contig_21630 | CCAGATAATTCAAGAGATG | AGTCTAATCGGTCTATATG |
| BB_Contig_1762 | TAATGAAGCAAGGCACTA | AATACTCCACACGATACC |
| BB_Contig_48951 | GTCCTCAGCACCAATCGT | GTTGTTGGAACTGTTGTTGATG |
| BB_Contig_1766 | TGAATGCTATAAGAATCGTA | ATTACCAATACACCAACAA |
| BB_Contig_17694 | GCCACTACTATAACAGAG | ATTACCTCCAAGATTGAAT |
| BB_Contig_49102 | AATTACAGTGTGCCAATGA | ACAACATCCTGACAGTCT |
| Ribosomal protein | AATTACAGTGTGCCAATGA | ACAACATCCTGACAGTCT |
| *Primers used for kdr detection* | | |
| BBPara1 | AACCTGGATATACATGCCTTCAAGG | TGATGGAGATTTTGCCACTGATG |
| BBPara3 | GGAATTGAAGCTGCCATGAAGTTG | TGCCTATTCTGTCGAAAGCCTCAG |
| *Primers used for RACE* | | |
| BB3-1 | TGATTCACAAGGTTCTCGGGCTCA |  |
| BB3-2F | ACCCAGAAGTGCAGAAGAAGCTCA |  |
| BB3-1R | TTGATGTCCATCCCAACGGGTACA |  |
